# Metabolic impact of polyphenol-rich aronia fruit juice mediated by inflammation status of gut microbiome donors in humanized mouse model

**DOI:** 10.3389/fnut.2023.1244692

**Published:** 2023-09-01

**Authors:** Stephanie M. G. Wilson, Jesse T. Peach, Hunter Fausset, Zachary T. Miller, Seth T. Walk, Carl J. Yeoman, Brian Bothner, Mary P. Miles

**Affiliations:** ^1^Department of Food Systems, Nutrition, and Kinesiology, Montana State University, Bozeman, MT, United States; ^2^Department of Chemistry and Biochemistry, Montana State University, Bozeman, MT, United States; ^3^Department of Research Centers, Montana State University, Bozeman, MT, United States; ^4^Department of Microbiology and Cell Biology, Montana State University, Bozeman, MT, United States; ^5^Department of Animal and Range Sciences, Montana State University, Bozeman, MT, United States

**Keywords:** gastrointestinal microbiome, high fat diet, inflammation, functional food, metabolomics

## Abstract

**Background:**

The *Aronia melanocarpa* fruit is emerging as a health food owing to its high polyphenolic content and associated antioxidant activity. Antioxidant-rich foods, such as Aronia fruit, may counter inflammatory stimuli and positively modulate the gut microbiome. However, a comprehensive study characterizing the impact of Aronia fruit supplementation has not been completed. Therefore, we completed analyses measuring the metabolic, microbial, and inflammatory effects of a diet supplemented with Aronia fruit juice.

**Method:**

Humanized mice were generated by colonizing gnotobiotic mice with microbiomes from human donors presenting disparate inflammation levels. Blood and fecal samples were collected throughout the course of an 8-week dietary intervention with either Aronia juice or a carbohydrate-matched beverage alone (2 weeks) or in combination with a high-fat diet to induce inflammation (6 weeks). Samples were analyzed using 16S rRNA gene sequencing (stool) and liquid chromatography-mass spectrometry (serum).

**Results:**

We demonstrated transfer of microbiome composition and diversity and metabolic characteristics from humans with low and high inflammation levels to second-generation humanized mice. Aronia supplementation provided robust protection against high-fat diet induced metabolic and microbiome changes that were dependent in part on microbiome donor. Aronia induced increases in bacteria of the *Eggerthellaceae* genus (7-fold) which aligns with its known ability to metabolize (poly)phenols and in phosphatidylcholine metabolites which are consistent with improved gut barrier function. The gut microbiome from a low inflammation phenotype donor provided protection against high-fat diet induced loss of microbiome β-diversity and global metabolomic shifts compared to that from the high inflammation donor.

**Conclusion:**

These metabolic changes elucidate pathway-specific drivers of reduced inflammation stemming from both Aronia and the gut microbiota.

## 1. Introduction

Chronic low-grade inflammation is a pathological characteristic of obesity-related conditions such as metabolic syndrome, type 2 diabetes mellitus, and cardiovascular disease and is noted by increased and sustained systemic levels of proinflammatory cytokines with no resolution (1–3). The accumulation and pro-inflammatory activation of macrophages in expanding adipose tissue, particularly around the abdomen, is considered a major contributor to chronic low-grade inflammation and promotes a detrimental shift in glucose and lipid metabolism (4–6). In addition to central adiposity, altered glucose metabolism, lipid metabolism, and hypertension are key components of metabolic syndrome, which proceeds and underlies many major chronic diseases (7). As an early driver of metabolic syndrome and chronic disease progression, intervention strategies are needed to counter inflammatory stimuli and chronic low-grade inflammation in metabolically at-risk populations.

The composition of, and host interactions, with the gut microbiota are of growing research and clinical interest. The gut microbiota comprises a diverse group of bacteria (and other microbes) in the human gastrointestinal tract that is altered in metabolic states associated with obesity and related conditions (8–11). A strong body of evidence in human and animal models supports the contribution of the gut microbiota to obesity development through increased energy harvest (8–10, 12–14). The composition and functionality of the gut microbiota, and ability to influence the host, is largely dependent on genetic and environmental factors (15, 16). Host diet, as a modifiable environmental factor, has been shown to influence gut microbial composition (16–19). For example, a high-fat diet (HFD) is often utilized in animal models to promote obesity and has been shown to concomitantly promote inflammation in the colon, adipose tissue, skeletal muscle, and the liver (20–22). Increased gut barrier permeability has been proposed as the connection between the gut microbiota and host inflammation. Alterations in gut bacteria, mucus bilayer composition, intestinal epithelial cells tight junctions, and local immune cells can all impact permeability (23–25). Chronic exposure to dietary fat is another factor that may negatively impact tight junction proteins and mucosal layer integrity, promoting increased translocation of bacterial constituents into the lamina propria and a subsequent induction of inflammatory responses establishing a positive feedback loop (23, 24, 26). Thus, frequent perturbations to barrier function can have profound clinical implications in diseases with localized inflammation and may partially explain a rise in systemic inflammation.

To combat inflammation, incorporation of functional foods may serve as a beneficial dietary strategy for metabolically at-risk populations to reduce the onset and progression of chronic disease (27, 28). Polyphenols can act as antioxidants which reduce cellular damage by free radicals and alter cellular signaling affecting inflammatory gene expression (29). A majority of polyphenols reach the large intestine and are subject to bacterial degradation into simpler phenolic metabolites (30). Polyphenols and phenolic derivatives can positively impact gut barrier integrity which may impact downstream proinflammatory processes (31) but may exert anti-inflammatory properties systemically (32). Importantly, polyphenols and their derivatives can modulate microbial diversity (33) and in turn, microbial metabolism can influence their bioavailability (34–36).

The black chokeberry, or *Aronia melanocarpa*, is of particular interest as an antioxidant-rich food. This hardy shrub produces fruit with the highest known antioxidant capacity for fresh fruit as measured by oxygen radical absorbance capacity (37). Commercial varieties are derived from a cross between *Aronia melanocarpa*, which is native to the United States, and European Mountain Ash (*Sorbus aucuparia*) sometimes classified as *Aronia mitschurinii* (38). *Aronia melanocarpa* fruit has a high polyphenolic content (39) with almost four times the anthocyanin content of blueberries, a lauded natural antioxidant-rich food (40). The antioxidant capacity of Aronia fruit (technically a pome fruit, like apples) stems from anthocyanins, procyanidins, and hydroxycinnamic acids. Aronia polyphenols have been previously shown to reduce inflammatory stimuli (41–45), the expression and concentration of pro-inflammatory cytokines (43, 44, 46, 47), and influence the colonic environment (45).

Dietary strategies, such as increased antioxidant availability through consuming polyphenolic-rich foods, present possible targeted therapeutic avenues for early chronic disease prevention. Dietary patterns can modulate gut microbiota composition and function, which are important in mediating host responses through production of bioactive compounds. Therefore, we hypothesized that compounds in host systemic circulation may provide key insights into the dynamic interplay between the gut microbiome and the host. In this study, we recruited healthy but metabolically at-risk human subjects and assessed their metabolic and systemic inflammatory profile. We created a humanized mouse model of chronic low-grade inflammation by transplanting stool from a human donor with high or low systemic inflammation into germ-free mice to account for variability in host genetics and host gut microbiota. We then introduced Aronia or control juice to their offspring and added a HFD as an inflammatory stimulus. Using advanced analytical measurement techniques including 16S rRNA gene sequencing and ultra-high performance liquid chromatography mass spectrometry, we investigated whether ingestion of polyphenolic rich Aronia juice could offer protective effects against HFD, and if that protection is dependent on gut microbial alterations including the inflammation phenotype of the human donors.

## 2. Methods

### 2.1. Experimental models and study participant details

#### 2.1.1. Human participants

Human participants for this paper were two adult women selected from a larger cohort of human participants (*n* = 40) that were metabolically profiled and assessed for resting and postprandial inflammation in response to a high-fat meal challenge. Potential subjects were recruited via advertisement between March 2016 to June 2018 and screened over the phone for eligibility. Inclusion criteria included being between 18 and 55 years old and having a body mass index between 27 and 36 kg/m^2^. Criteria for exclusion included antibiotics within 90 days of study enrollment, regular use of anti-inflammatory medications, use of estrogen-only contraceptives, wheat and/or dairy allergies or intolerances, were pregnant, or had any musculoskeletal, cardiovascular, gastrointestinal, or immunological condition that could interfere with the study. All potentially eligible subjects were screened over the phone for inclusion and exclusion criteria. The human subjects’ protocol was approved by the Institutional Review Board at Montana State University. Written informed consent was obtained prior to participation. The study was retrospectively registered October 2019 at ClinicalTrials.gov (NCT04128839). Forty overweight and obese men and women participated in testing of anthropometric and changes in serum metabolic and inflammatory markers during a high-fat meal challenge with a 50 g oral fat load and stool.

Subjects were categorized as having high fasting inflammation (HI) if they were above the group median at baseline in at least 4 of the 6 cytokines. Subjects were considered to have low fasting inflammation (LO) in they were above the group median in two or less cytokines. Individuals above the group median in 3 cytokines were considered neither HI nor LO. Fasting phenotype separation was confirmed by a two-sample *t*-test of each cytokine measure. Using a k-means analysis, the 40 participants were also grouped based on postprandial cytokine concentrations in response to an oral fat tolerance test (48). This analysis determined that the cohort contained three distinct inflammation groups based on cytokine levels. Groups were labeled as low-responders (LO), mid-responders (MID) and high-responders (HI). To isolate the impact of the gut microbiome on inflammation, each of the two participants selected as stool donors for the gnotobiotic mouse experiments was either LO or HI for both fasting and postprandial inflammation and both were as similar as possible for other factors that may influence inflammation (sex (both female), body fatness, waist circumference). Additionally, stool donors were 52 and 34 years of age, non-Hispanic Caucasian, and did not meet the criteria for Metabolic syndrome (additional information about donors found in Table 1).

**Table 1:** Anthropometric and metabolic characteristics of inflammation groupings and of selected human stool donors.

	Baseline inflammation			Inflammation response				Donors (*n* = 2)	
	Low (*n* = 19)	High (*n* = 16)	*p*-value	Low (*n* = 17)	Middle (*n* = 14)	High (*n* = 9)	*p*-value	Low	High
Women/Men	12/7	9/7	0.63	8/9	5/9	3/6	0.53	1/0	1/0
Age (years)	36.4 ± 10.5	35.3 ± 10.3	0.72	39.2 ± 9.6	34.8 ± 10.7	33.4 ± 9.8	0.3	52	34
Body mass index (kg/m^2^)	30.3 ± 1.9	30.6 ± 2.0	0.91	30.8 ± 2.6	30.1 ± 1.3	30.2 ± 1.6	0.67	27.7	35.9
Fat mass (%)	35.0 ± 5.9	36.7 ± 7.8	0.73	35.7 ± 7.6	35.8 ± 6.7	36.8 ± 6.6	0.92	35.1	48.1
**Insulin resistance**									
HbA1c (%)	5.28 ± 0.25	5.22 ± 0.32	0.74	5.4 ± 0.2	5.2 ± 0.2	5.2 ± 0.4	0.06	5.1	5.4
Homeostatic model of insulin resistance	1.7 ± 1.3	3.3 ± 2.5	**0.04**	2.2 ± 1.2	3.3 ± 2.6	1.6 ± 1.4	0.21	1.3	2.4
**Metabolic syndrome**									
Presence/absence	4/15	5/11	**0.03**	4/13	3/11	3/6	**0.02**	0/1	0/1
Fasting glucose (mmol/L)	5.3 ± 0.3	5.5 ± 0.4	0.53	5.4 ± 0.3	5.4 ± 0.5	5.5 ± 0.3	0.88	5.4	5.2
Fasting triglyceride (mmol/L)	1.5 ± 1.0	1.7 ± 0.9	0.33	1.9 ± 1.2	1.3 ± 0.7	1.7 ± 1.2	0.34	2.4	2.6
Fasting cholesterol (mmol/L)	4.7 ± 0.8	4.6 ± 0.8	0.65	5.1 ± 1.0	4.4 ± 0.6	4.4 ± 0.7	**0.02**	5.9	5.5
Fasting high-density lipoprotein (mmol/L)	1.4 ± 0.4	1.3 ± 0	0.27	1.4 ± 0.3	1.4 ± 0.5	1.3 ± 0.5	0.89	1.6	2.0
Waist circumference (cm)	94.4 ± 7.6	96.2 ± 9.8	0.8	95.6 ± 11.2	95.2 ± 9.4	95.4 ± 5.3	0.99	92.3	90.3
Systolic blood pressure (mmHg)	117 ± 14	111 ± 11	0.27	116 ± 9	112.4 ± 16	110 ± 15	0.55	128	114
Diastolic blood pressure (mmHg)	78 ± 10	74 ± 7	0.33	78 ± 9	75 ± 11	72 ± 8	0.41	78	83

Metabolic syndrome criteria are based on the National Cholesterol Education Program Adult Treatment Panel III definition. Data represents mean and standard deviation. Difference in sex and metabolic syndrome presence proportion were determined by a 2-sample test (baseline inflammation) or 3-sample test (Inflammation Response) for given proportions. All other *p*-values were determined by analysis of variance. Bold values indicate *p*-values less than 0.05.

#### 2.1.2. Germ-free mice

Female germ-free (GF) C57BL/6 J mice, originally purchased from the Jackson Laboratory (Bar Harbor, ME) were housed and bred at the American Association for the Accreditation of Laboratory Animal Care-accredited Animal Resource Center at Montana State University. The research protocol was approved by the IACUC at Montana State University. Mice were held in individually ventilated cages with sterile bedding before and after fecal transplantation from selected human stool donors. Two female mice received an inoculation with fecal material from a human donor categorized as having low or high systemic inflammation based on serum levels of six proinflammatory cytokines. Human donor stool slurry aliquots were administered to GF mice through oral gavage. Sexually mature male GF C57BL/6 J mice were added to each cage approximately 1 week after transplantation. Male mice removed prior to birth of pups. Pups from the inoculated dams were co-housed by sex (3–5 mice/cage) with different microbial inoculations placed in separate isolators. Mice from each microbial inoculation were assigned to one of two juice groups: Aronia (ARO_LO_, = 3, ARO_HI_, *n* = 5), or a sugar-matched juice (CON_LO_, *n* = 3, CON_HI_, *n* = 3).

### 2.2. Method details

#### 2.2.1. Human subjects protocol

Testing for the human subject cohort in this study took place in the Nutrition Research Laboratory at Montana State University. Anthropometric measurements were made, blood was collected after an overnight fast and following consumption of a high-fat meal for inflammation and metabolic status assessment, and stool samples were collected for analysis of gut microbiome composition. Two participants were selected from this pool as stool donors to humanize mice for the germ-free mouse experiments.

##### 2.2.1.1. Anthropometrics

Measurements were collected from subjects using the validated segmental multifrequency bioelectrical impedance analysis (SECA mBCA 515, Germany) (49). Subjects were instructed to refrain from eating, drinking, or exercising in the 3 h prior to testing. Fat mass (%) and estimated visceral adipose (liters) were used for descriptive analysis.

##### 2.2.1.2. Blood sampling

Subjects were instructed to avoid alcohol consumption and strenuous physical activity in the 24 h before their visit and to complete an overnight fast (10–12 h) before blood collection. Participant fasting blood samples were collected from the antecubital vein by a certified nurse or physician in the morning (6:30–8:30 a.m.). Blood was collected into 8.5 mL endotoxin-free serum separating tubes and allowed to clot for 15 min at room temperature before centrifugation (3,000 rpm, 15 min). Serum aliquots were frozen at −80°C until analysis.

##### 2.2.1.3. Metabolic syndrome markers

According to the National Cholesterol Education Program Adult Treatment Panel III definition, metabolic syndrome is the co-occurrence of insulin resistance, excess central adiposity, dyslipidemia, and hypertension (7). Blood markers of metabolic syndrome were determined from whole blood run on Picollo Xpress Chemistry Analyzer lipid panels (Abaxis, CA, United States) and included high density lipoprotein, triglyceride, and glucose. Blood pressure was taken in the morning after subjects had been seated for at least 15 min. Waist circumference, as an indicator of central adiposity, was taken in conjunction with anthropometric testing.

##### 2.2.1.4. Insulin resistance

Elevated glycated hemoglobin HOMA-IR are early indicators of insulin resistance. Glycated hemoglobin was determined using the Affinion2 analyzer (Abbott,) performed according to manufacturer instructions. Insulin was determined through ELISA (MP Biomedicals, United States) performed according to manufacturer instructions, with the average used for analysis. Fasting blood glucose and insulin were used to determine HOMA-IR according to the original HOMA-IR formula (50).

##### 2.2.1.5. Inflammation

A multi-cytokine approach was used for determination of fasting systemic inflammatory profile. Cytokine measurement was performed on serum samples using high-sensitivity multiplexing technology (Bio-Rad Bio-Plex^®^ 200 HTS) following procedures by Millipore (EMD Millipore Corporation, MA, United States). Classic systemic pro-inflammatory cytokines included granulocyte macrophage colony stimulating factor (GM-CSF), interleukin (IL)-1β, IL-6, tumor necrosis factor (TNF)-α. Interleukin-17 and IL-23, both of which serve a pro-inflammatory and regulatory role in the gut mucosa, were also measured. Human serum samples at each time point during the high-fat meal challenge were run in duplicate with the mean used for analysis.

##### 2.2.1.6. Stool sample collection

Collection kits were provided, and subjects were asked to follow printed instructions to self-collect a stool sample in the 24 h before their blood collection visit. After initial collection into a sterile disposable commode, a small portion of the sample was transferred into a sterile 50-mL conical vial and refrigerated until transportation to researchers. Samples were processed in an anaerobic chamber (Coy) with pre-reduced phosphate buffer saline and aliquoted into cryogenic vials at −80°C until analysis.

#### 2.2.2. Germ-free mouse experimental protocol

##### 2.2.2.1. Gut microbiota transplant and colonization

Two stool donors were selected based on their inflammation profile which occurred prior to gut microbial community profiling.

##### 2.2.2.2. Aronia juice targeted metabolomics LCMS and NMR analysis

Aronia juice was a blend of Mackenzie, Viking, and Autumn Magic cultivars grown at the Western Agricultural Research Center in Corvallis, Montana. Processed Aronia juice was examined to determine both the phenolic and carbohydrate composition. For quantifying the phenolic content, a targeted LCMS method was developed. The selected method was developed for use on an Agilent 6,538 quadrupole-time of flight (Q-TOF) mass spectrometer (Agilent, CA, United States) and an Agilent 1290 ultrahigh performance liquid chromatography system (Agilent, CA, United States) located at the Montana State University Proteomics, Metabolomics and Mass Spectrometry Facility. Separation was achieved using an Acquity HSST-3 UPLC reverse phase column, 1.8 μM, 100 mm/2.1 mm (Waters, MA, United States). The novel method was 18 min in length and consisted of HPLC grade water and acetonitrile (Fisher, MA, United States), both with 0.1% formic acid (Fisher, MA, United States), as mobile phases A and B, respectively. Analysis began with a flow rate of 0.3 mL/min and 95% A. At 2 min, the mobile phase composition changed linearly to 5% A at 15 min. A was held at 5% for 1 min and then a wash with 95% A was completed for the final 2 min of the run. The column compartment was kept at a constant 30°C throughout the analysis.

Standards were initially used to determine the retention time and peak area of nine phenolic standards at five different concentrations. Standards included anthocyanins and procyanidins previously found in Aronia (43). The collected data was then used to generate standard curves. Juice samples were diluted with HPLC grade water, 1:50 juice:water, and placed in mass spectrometry vials. Juice blends were analyzed via the same LCMS method as the phenolic standards and the concentration of specific phenolics were determined in each juice sample.

Carbohydrate composition was also explored using nuclear magnetic resonance (NMR) analysis. Juice was diluted with sodium trimethylsilylpropanesulfonate (DSS) and placed in an NMR tube. Analysis was performed using a Bruker 600 MHz Avance III NMR spectrometer (Bruker, MA, United States) with a 600 MHz TCI (H-C/N-D05Z) LT Probe located at the Montana State University Nuclear Magnetic Resonance Core Facility. From generated data, a control juice matching carbohydrate concentration was formulated. Carbohydrate standards were purchased and added to the placebo juice for the control mice. Placebo juice was analyzed using NMR and confirmed to have identical carbohydrate concentrations as the Aronia juice. Concentrations of phenolic and carbohydrate concentrations in the final Aronia blend juice are provided in Table 2.

**Table 2:** Carbohydrate and polyphenol content within Aronia *melanocarpa* fruit *juice*.

Compound	Concentration
**Carbohydrate (mM)**	
Sorbitol	672.74
Fructose	433.40
D-glucose	435.89
**Polyphenol (μM)**	
Neochlorogenic acid	8987.59
Chlorogenic acid	8598.77
Cyanidin 3-arabinoside	1224.29
Cyanidin 3-galactoside	589.40
Quercitin 3-glucoside	127.45
Cyanidin 3-glucoside	66.92
Cyanidin 3-xyloside	42.57
Quercitin 3-rutinoside	33.47
Quercitin 3-galactoside	19.36

##### 2.2.2.3. Juice and diet administration

At baseline (T0), regular drinking water was replaced with ARO or CON juice to begin a two-week familiarization period with the juice. ARO mice received an unpasteurized blend of Aronia juice, and CON mice received the sugar-matched beverage containing water, sorbitol, glucose, and fructose. The mice were housed in cages with free access to their respective juice. During the familiarization period, all mice received standard chow (LabDiet 5013).

After the 2-week familiarization period (T2), mice began a 6-week high-fat diet, delivered *ad libitum* concomitant with juice consumption. The HFD (Teklad TD.96132) was chosen to induce obesity and present an inflammatory stimulus (51). The HFD mimics a Western style diet and consisted of 40.6% fat, 40.7% carbohydrate, and 18.7% protein and was particularly rich in sugars and trans-fatty acids. All chow provided was sterilized via autoclaving or irradiation. A total of 150 mL of juice was provided per cage each week. Juice was refilled three times each week.

##### 2.2.2.4. Murine sample collection

Fecal pellets were collected at baseline (T0), at HFD start (T2), after 2-weeks of HFD (T4), and at the end of the 6-week HFD (T8, Supplementary Figure S1). Stool samples were frozen at −80°C until bulk DNA extraction. Blood samples were collected at the same interval into serum separating tubes. Pellets and blood were collected between the hours of 10:00 and 14:00 during designated experimental time points. Whole blood was allowed to clot for 15 min before centrifugation at 1200 RPM for 15 min with resulting serum aliquoted and stored at −80°C until analysis. After T8 sample collection, mice were euthanized via rapid CO_2_ asphyxiation.

One mouse in the second-generation had substantial weight loss (≥20% starting body weight) in the first week of the experiment and was euthanized according to IACUC protocol, leaving ARO_HI_ with four mice in total. All other groups were steady throughout the experiment.

##### 2.2.2.5. Genomic DNA extraction 16S rRNA gene sequencing

Extraction of bulk bacterial DNA from fecal samples was performed using Powersoil^®^ DNA Isolation Kit (Mo Bio Laboratories, Inc.) and bead beating. Extracted DNA was stored at −80°C until analysis. DNA was shipped overnight to the University of Michigan, Michigan Microbiome Project for Illumina MiSeq amplicon sequencing of the 16S rRNA V4 region. After DNA quantification, V4 amplicon libraries were generated with dual-index barcoded primers, then by library purification, pooling, and MiSeq paired-end sequencing. Raw sequencing reads were processed and curated using MOTHUR software (Version 1.35.1) (mothur.org) following the MOTHUR standard operating procedure for the MiSeq platform (52). In short, paired-end reads were assembled into contiguous sequences and screened for length and quality. The remaining contigs were aligned to the SILVA ribosomal RNA database (Release 132), a comprehensive collection of aligned rRNA sequences. Potentially chimeric sequences were identified and removed using the UCHIME algorithm in MOTHUR. Taxonomic classifications were assigned using the Bayesian classifier of the Ribosomal Database Project. Non-target reads were removed, and operational taxonomic units (OTU) were assigned using VSEARCH distance-based clustering at the 97% similarity threshold.

#### 2.2.3. Metabolomic analysis

##### 2.2.3.1. Targeted and untargeted LCMS metabolomics analysis

Serum samples were removed from −80°C storage and allowed to thaw. 20 μL of thawed serum was removed and placed in a clean vial after which 80 μL of ice-cold acetone was added to precipitate protein. Samples were then stored at −80°C overnight to aid precipitation. The next day, samples were spun in a centrifuge for 10 min at 20,000×g. The resulting supernatant was removed and placed in a clean vial while the remaining protein pellet was discarded. The metabolite-rich supernatant was concentrated under negative pressure in a Concentrator Plus (Eppendrof, Hamburg, Germany) to dryness. Dried samples were stored at −80°C until ready for LCMS analysis, at which time the samples were reconstituted with 40 μL of MeOH:H_2_0 (51) and placed in a clean mass spectrometry vial.

Untargeted LCMS analysis was completed on the same system as the phenolic analysis, an Agilent 6538 MS coupled to an Agilent 1290 UHPLC. Initial sample separation was accomplished on an Acquity BEH-HILIC column, 1.7 μm, 2.1 mm/100 mm (Waters, MA, United States). A 15-min method was employed using water and acetonitrile, both with 0.1% formic acid, as mobile phases A and B, respectively. 45% A was held for the first 2 min, after which a linear gradient increased A until 11 min to 70%. 70% was held for two additional minutes until switching to 0% A at 13 min. The column compartment was kept at 40°C and the flow rate was 0.2 mL/min. MSMS, or tandem MS, was completed on the same system with the same LCMS settings to identify metabolites. Collision energies of 10, 20 30, and 40 V were used to fragment analytes. This method yielded over 1,000 metabolites from each sample.

A second targeted LCMS analysis was also undertaken, specifically to determine mouse serum concentrations of trimethylamine-N-oxide (TMAO), a compound associated with phosphatidylcholine (PC) levels, which were found to be variable in our untargeted results (53). Analysis was completed on the same instrument as previously described but with a different method. A 6-min targeted method was used with acetonitrile and 10 mmol/L ammonium formate as mobile phases A and B, respectively. A 10–40% A gradient was used over 6 min with a compartment temperature of 30°C (54). TMAO retention time and a standard curve was generated by analyzing authentic standards. After serum sample analysis, TMAO relative concentrations were integrated, and the concentrations were determined using MassHunter v11.0 (agilent.com).

### 2.3. Quantification and statistical analysis

#### 2.3.1. Human cohort characteristics

Descriptive statistics of the participant physical characteristics and metabolic profile by inflammation phenotype were performed by ANOVA in RStudio (V. 1.4.1106) running base R 4.2.2.

#### 2.3.2. Power analysis

A *post hoc* power analysis was utilized as an expected effect size for treatment differences was unknown. A power analysis was performed from alpha diversity data collected during the high fat diet using the G*Power3 program (55). As described in the figure four legend, significance was tested for the difference observed between high- and low-inflammation groups in Aronia-treated mice following high-fat diet (at week 8). Program inputs were as followed: effect size d of 7.99, alpha error probability of 0.05, group 1 size of 4, and group 2 size of 2. Based on these parameters, the power achieved was 1 with a noncentrality parameter delta of 9.17, critical t of 2.12, and observed t of 7.68.

#### 2.3.3. Gut microbiome data analysis

A total of 2,732,246 raw reads were obtained across all samples. To aid unbiased diversity matrices due to sequencing depth, data was randomly subsampled at the minimum number of sequences across samples. Subsampling resulted in a total of 2,082,652 high quality reads. Alpha diversity was calculated using *phyloseq* 1.38.0 (R) and visualized using effects plots from the R *effects* package. Beta-diversity analyses were performed on subsampled data with filtering of OTUs less than 3 counts in at least 20% of the samples. Permutational analysis of distance matrices with stratification by cage and 999 permutations was performed using the adonis function in the *vegan* package 2.5–6 (R). Canonical correspondence analysis (CCA) was used to assess the impact of time, donor, and juice treatment on the microbial community at the species level using *phyloseq* 1.38.0 (R). The contribution of variables in CCA was assessed through a permutation test with 999 permutations with cage stratification.

A linear regression framework for differential abundance analysis (LinDA) was applied from the *MicrobiomeSta*t R package. LinDA considers the correlated nature of the microbiome in longitudinal study designs which can be extended to the mixed effects model and is similar to ANCOM-BC but is different in that it does apply a bias correction and does not utilize the E-M algorithm (56). The response is abundance data transformed using the center log-ratio and applies a bias term from the compositional effect of the microbiome. The bias is corrected using the mode of regression coefficients, and *p*-values are generated from the bias-corrected regression coefficients and applies a multiple comparison correction. LinDA was used to assess baseline differences in microbial genera. LinDA was also used to assess changes over time from juice and from juice during HFD, with an interaction between treatment and donor and a random effect for the mouse number for mixed effects modeling. Default parameters for LinDA were applied to counts, aggregated at the genus level, except for the mean percentage of non-zeros cutoff which was set to 0.0001 and n.cores adjusted for the mixed effects models.

Microbial ecological analyses and visualizations performed in RStudio (V. 2023.3.0.386) running base R 4.2.2.

#### 2.3.4. LCMS and NMR data analysis

After MS analysis, data was converted to either a .mzML format for MS data and .mgf or .abf format for MSMS data using MSConvert v3.0 (proteowizard.sourceforge.io) and Reifycs Analysis Base File Converter (reifycs.com) (57). MS data was then interpreted using mzMine v3 (mzmine.github.io) and statistical analysis was completed using MetaboAnalyst v5.0 (metaboanalyst.ca) (58, 59). An in-house library was used based on authentic standards for level 1 identifications (60). Level 2A annotation was completed with spectral fragmentation matching using MSDial software v4.9 (61) and the MassBank spectral library (62). A 0.005 MS and 0.01 MSMS cutoff were used along with an 80% score match for positive annotations. Level 3 *in silico* annotation was also performed with SIRIUS software v5.0 (bio.informatik.uni-jena.de/software/sirius) by searching all biological databases using a 15 ppm error window (63). Results with a similarity score of only 60% and with the highest score were selected. NMR results were examined using Chenomix NMR Suite software v7.6 (chenomx.com).

## 3. Results

### 3.1. Gnotobiotic mice humanization

**Figure 1:**
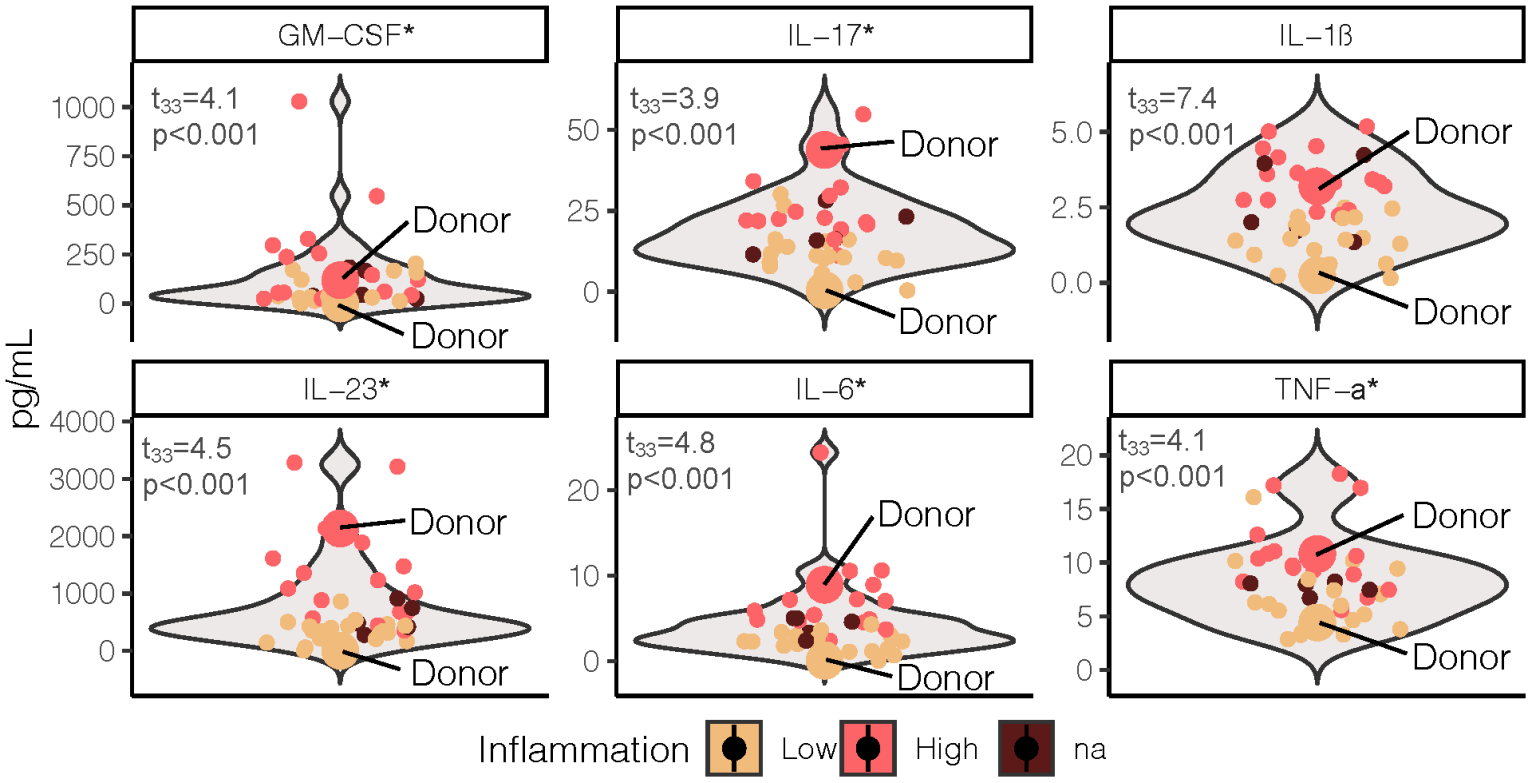
Fasting proinflammatory profile grouped by LO and HI inflammation phenotype. Test statistics and *p*-values were determined by two sample *t*-test with phenotype (excluding NA *n* = 5) as the grouping variable. *Indicates the cytokine values were log transformed to meet normality assumption. LO, low inflammation; HI, high inflammation; GM-CSF, granulocyte macrophage colony-stimulating factor; IL, interleukin; TNF, tumor necrosis factor.

**Figure 2:**
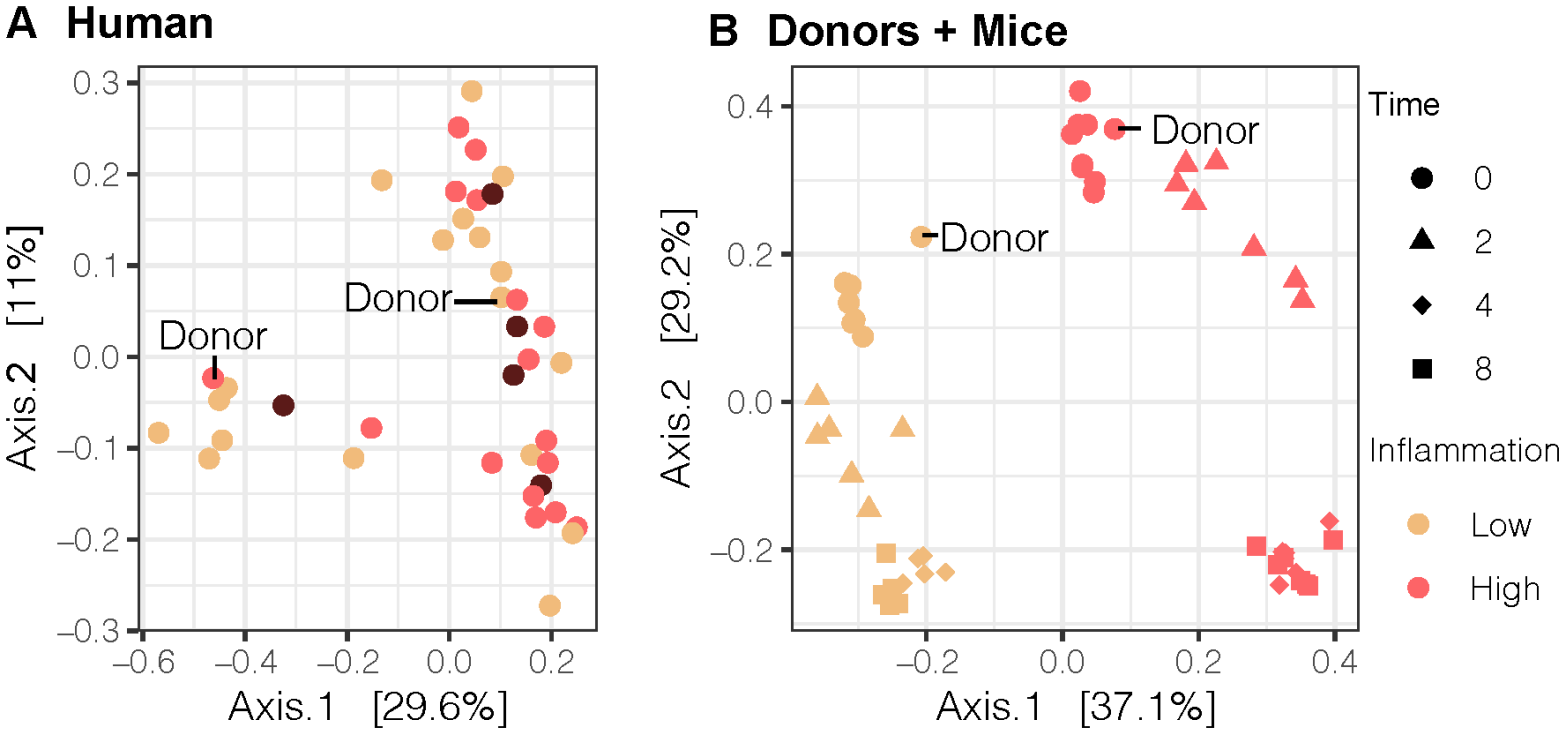
Bray-Curtis Principal Coordinate Analysis (PCoA) of the gut microbial community for the **(A)** human cohort (*n* = 40), **(B)** human donor compared to 2nd generation mice (*n* = 13). Our LO and HI INF donors are highlighted in **(A,B)**. PCoA results were plotted according to the first two components. Axes explain the percentage of variance in the gut microbial composition at the genus level between samples. LO, low inflammation; HI, high inflammation; INF, inflammation.

We sought to transfer an inflammation phenotype from human stool donors to gnotobiotic mice so that we could investigate the impact of antioxidant rich Aronia berries on both the gut microbiome and on serum metabolomic profiles. We recruited a human cohort between the ages of 18 and 55 with similar physical characteristics (Supplementary Figure S2). Analysis of this cohort revealed variation in inflammation, insulin resistance, and metabolic syndrome status (Table 1). Our inflammation phenotyping binned individuals in low- or high-inflammation (LO or HI) groups (Figure 1). From this cohort, one LO and one HI participant were selected as stool donors based on their fasting and postprandial inflammatory profiles. To isolate the impact of the gut microbiota, subjects with similar percent body fat, waist circumference, and sex were selected. Importantly, neither donor met the criteria for metabolic syndrome. The LO donor was LO in fasting inflammation and postprandial response to an oral fat load. Conversely, the HI donor was HI in fasting inflammation and postprandial inflammatory response. Further, 16S rRNA analyses indicated that the LO stool donor had a higher OTU richness (377 vs. 338) and Shannon Index (4.1 vs. 3.7) than HI. LO and HI donors also differed in β-diversity (Figure 2A).

With donors selected, stool samples were transplanted into gnotobiotic mice. We compared the gut microbial composition of pups from dams inoculated with different fecal microbiota transplant (FMT) human donors. Beta-diversity analysis using Bray-Curtis PCoA plots demonstrated that second-generation mice with direct exposure from their inoculated GF mice parent resembled their respective human stool donor (Figure 2B). Moreover, the microbial community was distinct by donor and remained distinct throughout the duration of the mouse experiment (*R* = 0.343, *p* < 0.001, ANOSIM_donor_). LO mice also differed in the abundance of multiple bacterial genera compared to HI mice at baseline as detected by the LinDA differential abundance analysis (Supplementary Figure S3).

**Figure 3:**
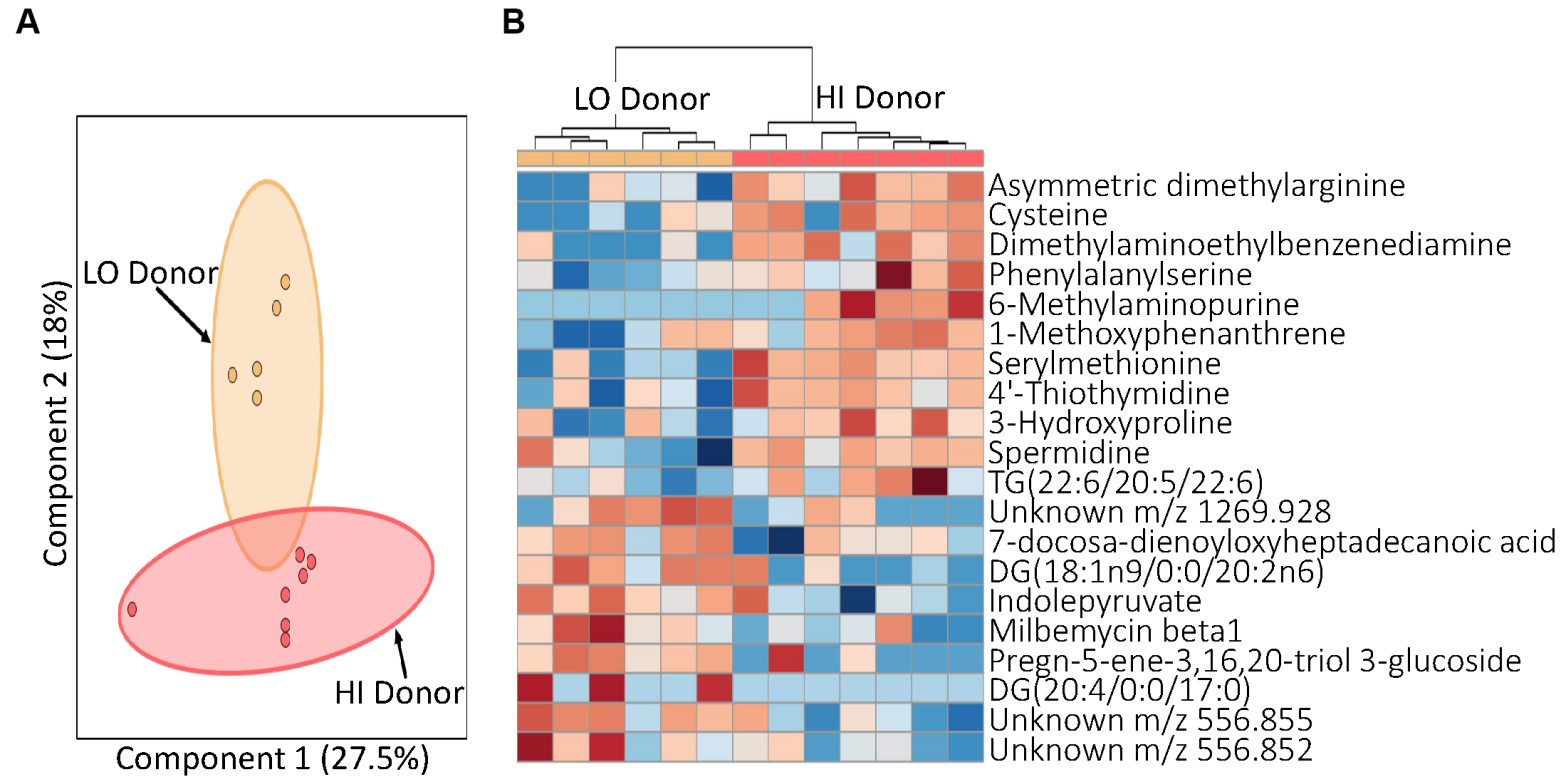
Profiling of serum metabolites in second generation mice prior to experimental treatment (T0). **(A)** A principal component analysis (PCA) by juice treatment with 95% confidence intervals shown. **(B)** Heatmap for T0 indicating top 20 metabolites. Distinct blocks of metabolites upregulated with both HI and LO inflammation donors are seen. Red, upregulation; blue, downregulation. LO, low inflammation; HI, high inflammation.

After confirming the transfer of LO and HI microbiomes to the mice, our next task was to determine how the respective microbiota influenced host metabolism. To do this, we examined the metabolomic profiles of serum from mice receiving the LO and HI stool transplants using liquid chromatography-mass spectrometry (LCMS). A global view of the collected LCMS data revealed different metabolic profiles in mice with LO and HI stool donors as seen in a 2-D principal component analysis (2D-PCA) (Figure 3A). This striking contrast between profiles is highlighted by the level of separation seen between samples with the same matrix, serum, and the high percentage of variation in component one and two with 27.5 and 18%, respectively. A focused examination of the metabolites that best differentiated the groups was also performed. LO mice showed upregulation of diacylglycerides and downregulation of metabolites such as cysteine 3-hydroxyproline relative to HI mice (Figure 3B). LO mice also demonstrated an increase in indolepyruvate, a secondary metabolite produced by specific microbes in the gut microbiome. Metabolomic profiling of LO and HI mice, taken with the microbial analysis, indicated that not only were the microbial communities of stool donors effectively transferred to second-generation pups by fecal microbiota transplant to germ-free dams, but this led to altered metabolic states that were also present in the human donors.

### 3.2. Aronia juice impact on the gut microbiome

**Figure 4:**
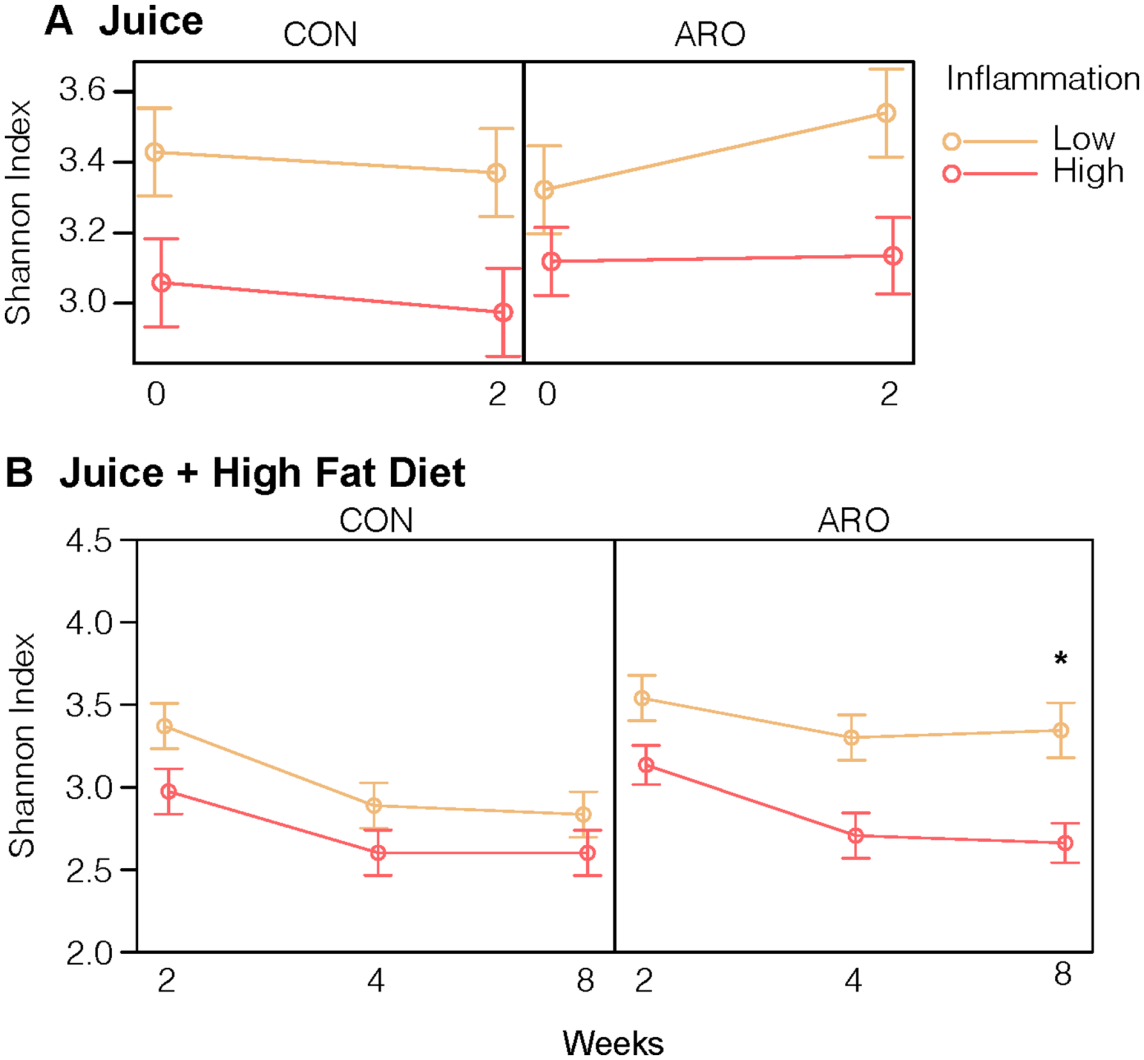
Shannon Index, an alpha diversity measure, of the gut microbial community in second-generation mice in response to **(A)** 2-Week juice treatment and **(B)** juice treatment and HFD. *Indicates a difference in means at the end of the HFD (Time 8) by donor and juice treatment. Points indicate group averages and bars represent 95% confidence intervals derived from the all effects function in the R *effects* package. ARO, Aronia juice; CON, control juice; LO, low inflammation; HI, high inflammation; INF, inflammation; HFD, high-fat diet.

Successfully transferring the gut microbiota and corresponding metabolic phenotypes positioned us to explore the impact of anthocyanins from Aronia juice (ARO) vs. a sugar matched control (CON) on the gut microbiome over time and to identify how LO and HI microbiota influenced alpha and beta diversity metrics and taxonomic responses to Aronia. As with the human donors, second-generation HI INF mice had a lower Shannon index, a measure of α-diversity, at baseline, on average compared to mice exposed to LO INF microbiome (β = −0.37, *p* = 0.001). Second-generation mice began a two-week acclimatization period with CON and ARO juice. We did not detect a three-way interaction between juice treatment, time, and donor (*p* = 0.30); however, a statistical main effect for ARO on α-diversity was observed with an average increase in the Shannon index of 0.28 (*p* = 0.045) after 2 weeks (Figure 4A).

Having established that ARO differentially impacted community diversity metrics based on the donor microbiota, our next step was to determine how mice with the LO and HI gut microbiota and metabolic phenotypes responded to an inflammation challenge. We presented a 6-week high-fat diet (HFD) to induce obesity and inflammation in combination with ARO and CON treatments in both LO and HI mice. With the introduction of a HFD, α-diversity declined with CON irrespective of FMT donor microbiome exposure (Figure 4B). ARO juice provided protection from HFD-induced loss of α-diversity in second-generation mice with a LO INF microbiota (*p* = 0.02). HI mice which received ARO juice displayed the same decline in α-diversity as CON groups.

**Figure 5:**
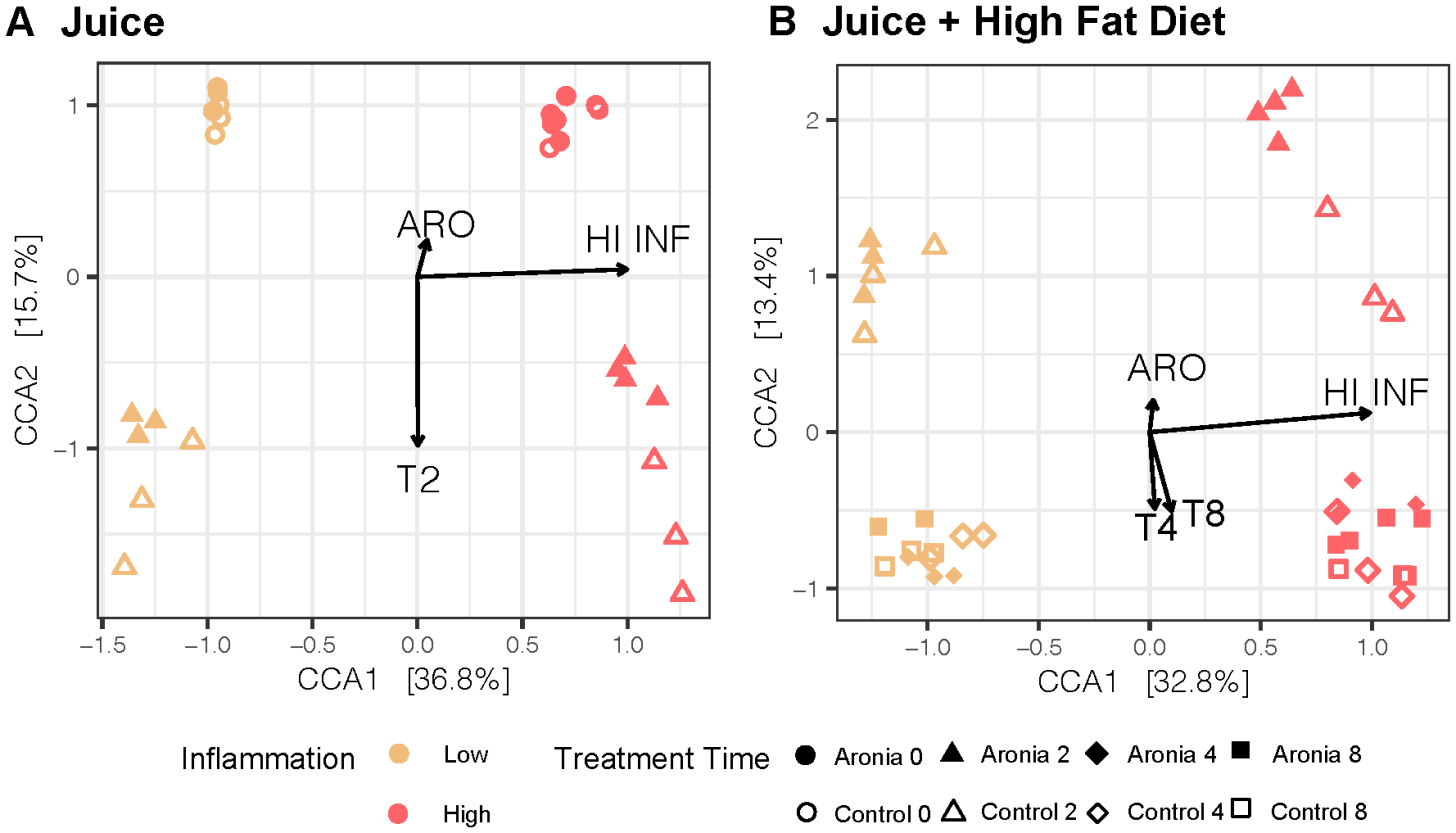
Canonical Correspondence Analysis in 2nd generation mice with treatment, time, and inflammation as constraining variables for the **(A)** first 2 weeks with juice and **(B)** 6-weeks with juice and HFD. CCA results were plotted according to the first two components. Axes explain the percentage of variance in the gut microbial composition at the genus level between samples. ARO, Aronia juice; LO, low inflammation; HI, high inflammation; INF, inflammation; T, time in weeks.

To extend our analysis of microbiome diversity, we performed a canonical correspondence analysis (CCA) to better understand the community from introduction of ARO and the eventual joint impact of juice and HFD. In Figure 5A, a distinct separation in the second-generation microbial community based on the original human FMT donor is observed (*F* = 34.0, *p* = 0.001). Additionally, the introduction of juice promoted a similar shift in β-diversity that was donor-independent (*F* = 4.5, *p* = 0.001). However, mice which received ARO had a smaller shift in β-diversity over 2 weeks relative to mice receiving CON (*F* = 4.2, *p* = 0.001). A large shift in β-diversity was seen after only 2 weeks on a HFD (*F* = 9.0, *p* = 0.001) and was less pronounced in LO vs. HI mice (*F* = 6.7, *p* = 0.001) (Figure 5B). A modest Aronia effect on β-diversity was observed across LO and HI and was counter in action with the HFD (*F* = 2.7, *p* = 0.004).

**Figure 6:**
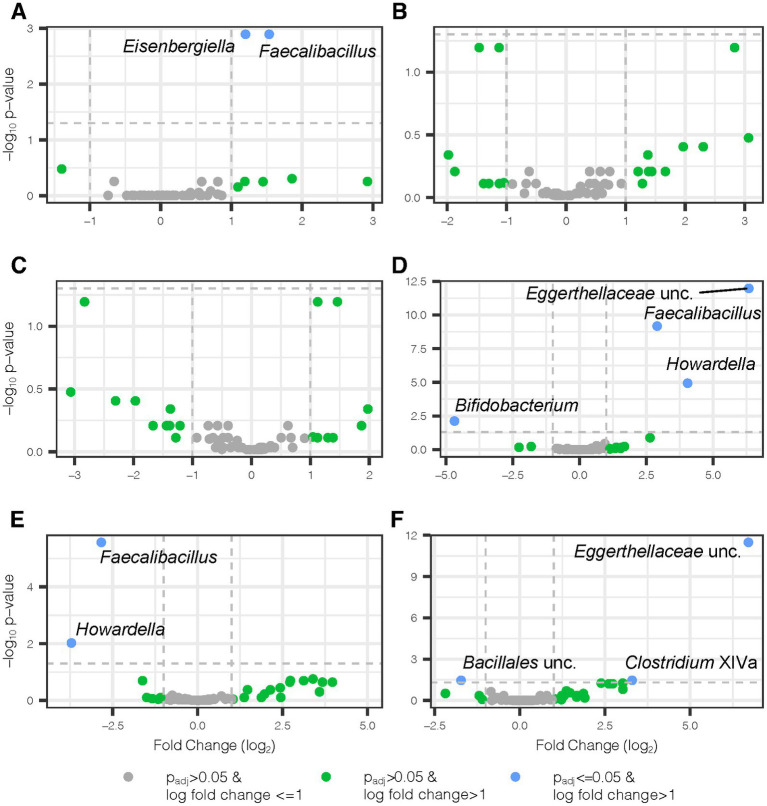
Differential bacterial genera with Aronia juice and donor-dependent changes. Volcano plots show differential genera detected by two separate LinDA analyses, one for weeks 0–2 and a second for weeks 2–8. In weeks 0–2, mice received standard chow and either Aronia or control juice. In weeks 2–8, mice switched to a high fat diet and continued their existing juice treatment. All juice comparisons highlight Aronia juice in comparison to control juice. **(A)** Juice main effect from weeks 0–2, **(B)** juice and donor interaction effect from weeks 0–2 where donor comparison is LO vs. HI inflammation mice, **(C)** juice and donor interaction effect from weeks 0–2 where donor comparison is HI vs. LO inflammation, **(D)** juice main effect from weeks 2–8, **(E)** juice and donor interaction effect from weeks 2–8 where donor comparison is LO vs. HI inflammation, and **(F)** juice and donor interaction effect from weeks 2–8 where donor comparison is HI vs. LO inflammation. padj, Benjamini-Hochbeg adjusted *p*-value; lfc, log fold change; unc, unclassified.

Shifting from measures of microbiome diversity to individual components, a HFD introduced microbial taxonomic changes in second-generation mice that were donor-independent and donor-dependent. We applied LinDA, a differential abundance analysis method which corrects for the correlated nature of the microbiome in repeated measures designs, to assess genus level changes in the gut microbiota from the addition of juice and the influence of juice concomitant with HFD. We observed a 1.2 and 1.5-fold respective increase in the abundance of *Eisenbergiella* and *Faecalibacillus* in mice receiving Aronia juice relative to the mice receiving control juice (Figure 6A). We did not observe donor-dependent changes from the juice supplementation period as indicated by a lack of differentially abundant bacterial genera in Figures 6B,C. With detected several differential taxa with Aronia juice after 6 weeks of a HFD, including increased abundance of *Faecaelbacillus, Howardella*, and an unclassified genus within the *Eggerthellaceae* family within mice on Aronia juice compared to mice on control juice (Figure 6D). The greatest taxonomic change with Aronia during the experiment was seen in the unclassified *Eggerthellaceae* genus with an increase of 6.3-fold compared to control juice. However, when the donor and juice interaction is considered, it was found that the *Faecalibacillus* and *Howardella* decreased in abundance with Aronia juice in LO mice (Figure 6E). A substantial increase in n unclassified *Eggerthellaceae* genus was detected in HI compared to LO inflammation mice (Figure 6F). Additionally, unique to HI inflammation mice was an increase in the abundance of *Clostridium* Cluster XIVa and an unclassified member of *Bacillales* decreased. Overall, our microbial analysis indicated that Aronia juice offered a protective effect against community shifts from HFD and that a small number of select genera were differentially abundant in response to juice.

### 3.3. Aronia juice impact on metabolomic profiles

**Figure 7:**
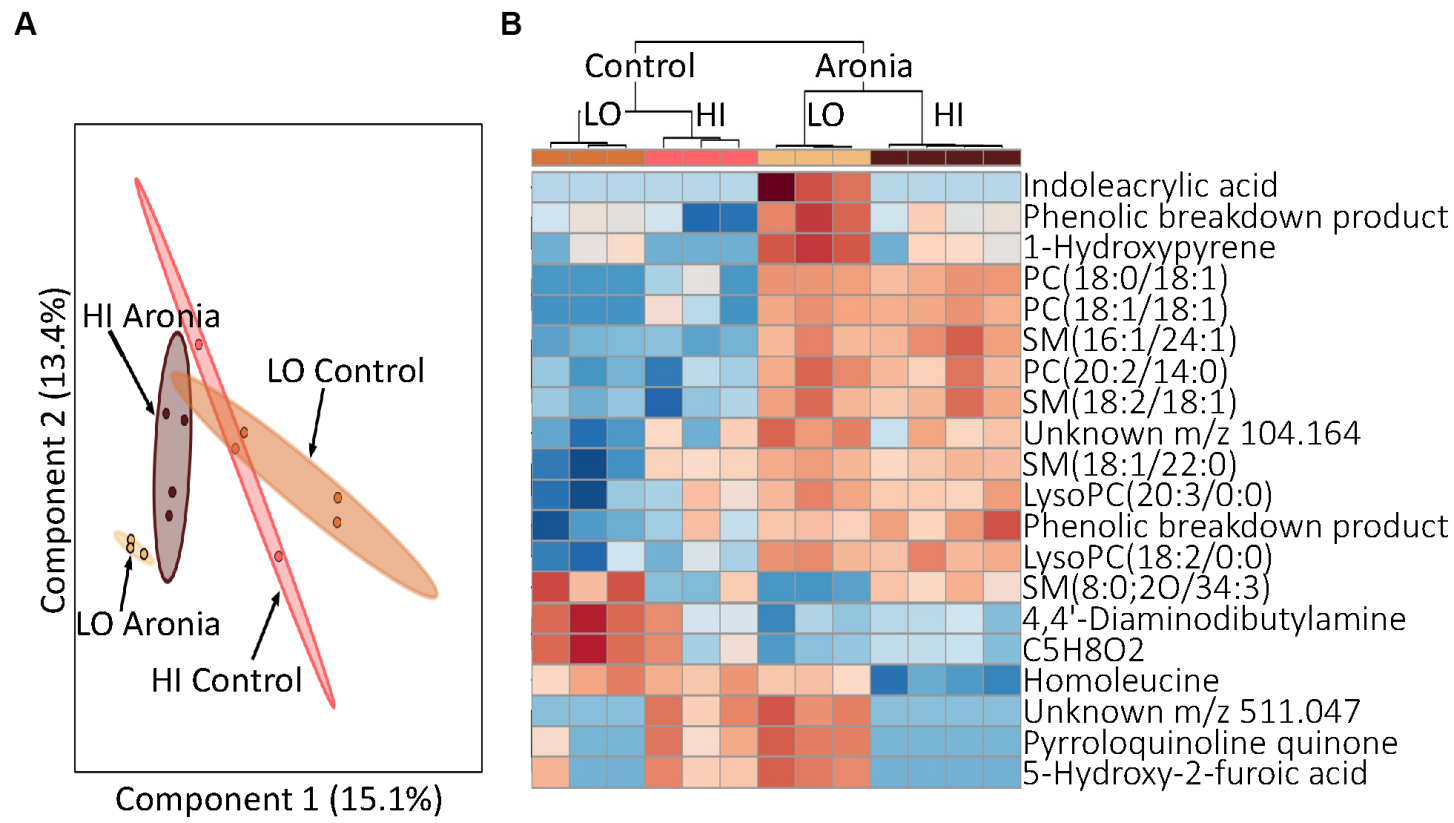
Profiling of serum metabolites in second generation mice after 2 weeks of ARO and CON treatment (T2). **(A)** A principal component analysis (PCA) by donor and juice treatment. **(B)** Heatmap for T2 indicating top 20 metabolites. Dendrogram indicating separation by juice treatment followed by donor type. Red, upregulation; blue, downregulation. LO, low inflammation; HI, high inflammation, PC, phosphatidylcholine; PE, phosphatidylethanolamine; SM, sphingomyelin; LysoPC, lysophosphatidylcholine; PA, phosphatidic acid.

To determine how LO and HI INF microbiota influence the impacts of Aronia juice, our final aim was to analyze samples via LCMS and determine the effect of ARO vs. CON on metabolomic profiles. Although the metabolic profiles of initial serum samples grouped by LO and HI donor (Figures 3A,B); the effect of ARO supplementation was evident by week two (Figure 7A). Week two samples showed definitive grouping based on ARO vs. CON juice first, followed by donor (Figure 7B). A closer inspection of discriminative metabolites between juices at week two indicates altered lipid metabolism in ARO mice relative to CON mice. Specifically, phosphatidylcholines (PCs) and sphingomyelins (SMs) were shown to be upregulated in ARO mice.

**Figure 8:**
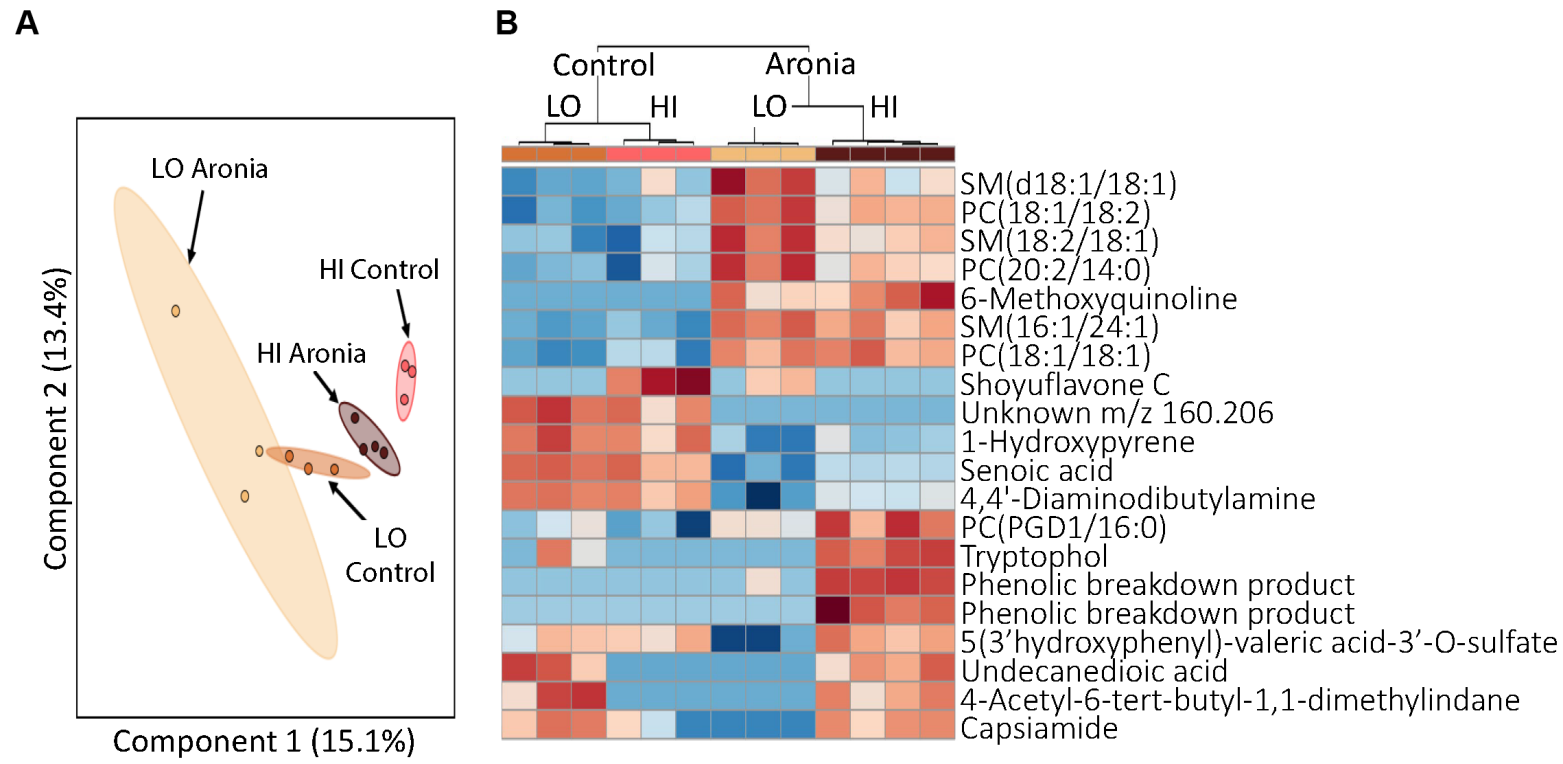
Profiling of serum metabolites in second generation mice after 8 weeks of experimental treatment (T8). **(A)** A principal component analysis (PCA) by donor and juice treatment. **(B)** Heatmap for T8 showing top 20 discriminating metabolites. The dendrogram at the top shows grouping first by juice treatment followed by donor group. Red, upregulation; blue, downregulation. LO, low inflammation; HI, high inflammation, SM sphingomyelin; PC, phosphatidylcholine.

Finally, having demonstrated that LO and HI INF gut microbiota and ARO treatment interact and affect metabolism, we sought to determine whether the LO microbiota and or ARO confer protection against HFD-induced inflammation alone or in combination with each other. The HFD led to dramatic changes in metabolic profiles that superseded the effects of both the microbiome and the juice treatment. A heatmap of discriminating metabolites in week 2 and week 4 demonstrates the impact of the high-fat diet (Supplementary Figure S4). Metabolites were found to group first by time, then by treatment and finally by donor. Upregulation of SMs and PCs discriminated pre- and post-HFD metabolomic profiles. Week 8 was the final time point in our study. Analysis of these final samples revealed that both juice and donor had a significant influence on metabolomic profiles. Although the impact of juice and donor were intertwined, disparate metabolomic profiles were found for both LO and HI INF microbiota and ARO vs. CON treatment. A PCA revealed the effects of the donor were still prevalent after 8 weeks of experimental juice and the introduction of a HFD (Figure 8A). However, a closer examination of the discriminating metabolites between groups indicated the impact of juice supplementation. In this analysis, ARO correlated with an upregulation of specific PCs and SMs (Figure 8B). The donor effect was also still seen as HI and LO clusters of upregulated metabolites were found.

**Figure 9:**
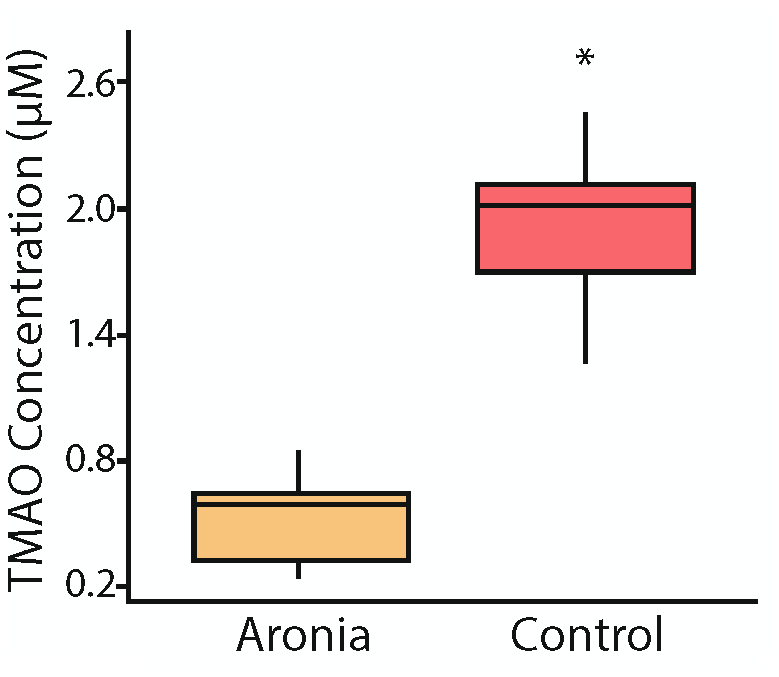
Serum trimethylamine-N-oxide (TMAO) concentrations after 8 weeks (T8). Concentrations of TMAO in mouse serum after 8 weeks of juice treatment indicating significantly lower levels in mice which received Aronia fruit juice compared to those that received control juice. A *t*-test comparison indicated a *p*-value less than 0.05 (*).

Due to the continued variability seen in PC concentrations between treatment groups, TMAO concentrations were investigated. TMAO is a product of choline metabolism and a proposed marker of cardiovascular risk (64). TMAO is produced in the liver from trimethylamine, a secondary metabolite derived from microbial catabolism of choline. As breakdown of the choline found in PCs increases, TMA availability and, therefore, TMAO concentration increase. TMAO concentrations were found to be lower (*p* < 0.001) in the Aronia supplemented mice at the conclusion of the study (Figure 9).

## 4. Discussion

Using gnotobiotic mice, we were able to elucidate the impact of Aronia juice supplementation on the gut microbiome and the serum metabolome before and during a high-fat diet. Success in this study relied heavily on our ability to humanize mice by transferring microbiomes from human stool donors who were metabolically similar but had a different inflammatory profile as well as the ability to transfer the donor microbiome to second-generation pups. Additionally, we were able to generate unique metabolomic profiles from each donor specific mouse population. By exploring the beta diversity of the microbiomes along with the grouping of the global metabolomes and specific microbial populations and metabolites, our analysis indicated that two distinct humanized mouse populations were created. This is a significant observation that has broad impacts on the use of animal models to study inflammation.

Each mouse population had a unique microbial profile that closely resembled that of the original human stool donor (Figure 2). Human stool donors were metabolically similar (Table 1), with the specific donors selected based on their distinct low and high systemic inflammatory profile. A higher microbial diversity was observed in the selected human LO microbiome profile vs. HI microbiome profile, a trait that was recapitulated in the mice and helped separate the microbial communities into two distinct groups at baseline (Figure 4). The transfer of microbiomes from inoculated germ-free mice to the second-generation without declines in diversity has been previously observed (19). Additionally, our findings echo previous research which found that low gut bacterial diversity in humans correlated with a pronounced inflammatory phenotype (65). High bacterial diversity is a generally accepted indicator of a healthy gut and is thought to contribute to the resistance of microbial communities to ecological perturbations and the ability to return to equilibrium (10). Along with distinct microbial communities, metabolomic analysis indicated discrete global profiles in each donor population. Metabolomic profiles of the mice grouped based on donor and revealed the presence of discriminating metabolites (Figure 3; Supplementary Table S2). One of discriminating metabolites, indolepyruvate, was upregulated in the LO mice and is only known to be produced by specific *Clostridium* species associated with the LO microbiome. Conversely, 3-hydroxyproline was upregulated in HI mice. 3-hydroxyproline is an imino acid shown to stimulate inflammation and modify macrophage signaling (66). 3-hydroxyproline has also been shown to be diet dependent in mammals, yet mice in this study received the same food, indicating a microbial influence promoting 3-hydroxyproline production (67).

After 2 weeks of juice supplementation and normal diet, a shift was detected in the gut microbiota of second-generation mice (Figure 5). The control and Aronia juice both contained glucose, fructose, and sorbitol, and the introduction of these carbohydrates was also marked by a pronounced shift in β-diversity after 2 weeks across both LO and HI mice. The impact of Aronia on β-diversity was minimal, though we detected a statistical main effect with Aronia juice for an increase in α-diversity (Figure 4). We observed that bacterial changes during the Aronia supplementation period were not donor-specific and that Aronia promoted an increase in the abundance of *Eisenbergiella* and *Faecalibacillus*. *Eisenbergiella* is negatively correlated with inflammatory gene expression (68) and one study has found *Faecalibacillus* to positively associate with hypertension (69). The metabolic capacity of either genus is still largely unknown; thus, additional research is required to better understand the metabolic capacity of these two bacterial taxa and particularly, their capacity to metabolize (poly)phenolic compounds and relationship to health outcomes.

Not only were changes in the gut microbiome makeup observed after 2 weeks of supplementation, but mouse metabolism was also altered as well. Prior to juice introduction mouse metabolic profiles had grouped by donor (Figure 3; Supplementary Table S2). However, by 2 weeks profiles indicated a significant shift and began to cluster by juice group (Figure 7; Supplementary Table S2). Indoleacrylic acid was upregulated with Aronia only in mice with the low inflammation gut microbiome donor. Indoleacrylic acid is a tryptophan catabolism metabolite with potent antioxidant, anti-inflammatory, and enhanced gut barrier function effects. This metabolite is only produced by three bacterial species of the *Peptostreptococcus* genus and one species of the *Clostridium* genus, which pinpoints the sources of this microbiome dependent health benefit of Aronia (70). Further, the metabolic switch was characterized by an increase in phosphatidylcholines (PC) in Aronia treated mice. Increased PC composition has been indicated in a variety of positive health benefits. In addition to their role as structural components of cellular membranes, PCs can counter LPS-induced inflammation by inhibiting tumor necrosis factor expression as well as limiting inflammatory responses by suppressing pro-inflammatory pathway signaling (71–73). These positive benefits are augmented by the ability of PCs to impact membrane composition and fluidity. Barriers in the intestinal mucus are ameliorated with higher PC composition and a decrease in PC composition is seen in inflammatory conditions such as inflammatory bowel disease and ulcerative colitis (74–76). This observation is likely due to PC-specific modulation of membrane composition and a PC-induced increase in membrane fluidity which contributes to normal cell function. Reduction in PCs has been indicated in rigid membranes and promoted aging processes (77–79). The increased serum PCs seen in Aronia treated mice may be due to complexation of phosphatidylcholines with polyphenolic compounds and polyphenolic derivatives. Anthocyanins are larger polyphenols which do not pass easily through the intestinal barrier by diffusion, thereby limiting bioaccessibility to the host. Formation of phyto-phospholipid complexes, which improves membrane permeability, miscibility, and absorption of polyphenols from the gut for host bioavailability (80–82). Given we did not observe phosphatidylcholine increases in control mice receiving the same diet, we propose that the increase in phosphatidylcholine with Aronia supplementation was facilitated through the complexation process of polyphenolic compounds present in Aronia juice.

To elicit an inflammatory response reflective of a Western diet, the normal diet (13.3% fat) was switched to a high-fat diet (41.7% fat). This allowed for a comparison of Aronia supplementation with and without an inflammatory stimulus and provided a model to determine the possible Aronia driven effects during an HFD. High fat diets induce reproducible shifts in the gut microbiome independently of fluctuations in body weight (83, 84). As expected, we observed a substantial global shift in the microbial community with the introduction of HFD across both donor groups (Figure 5B). This effect was donor-dependent as HFD facilitated a prominent shift in the β-diversity of the microbiota to which the LO INF mice were more resistant. Further, a key finding in this study was the donor-dependent protective effect of Aronia supplementation against a loss of α-diversity during the HFD, with LO INF mice less susceptible to losses in α-diversity (Figure 4B). A greater α-diversity and increased presence of beneficial taxa such as *Parasutterella*, *Faecalibacterium*, and *Clostridium* Cluster XIVa (Supplementary Figure S3) may have partially contributed to this protective effect in LO INF mice.

The mice continued the experimental juice for 6 weeks concomitant with HFD. After 6 weeks, additional juice-dependent changes in the gut microbiota were observed. The impact of Aronia supplementation on the global microbial composition during HFD was modest as indicated by our constrained ordination (Figure 5B). However, specific taxonomic groups were unique to Aronia supplementation, with a greater abundance of *Faecalibacillus*, *Howardella*, and an unclassified member of *Eggerthellaceae* during the last 6 weeks (juice and HFD). The unclassified *Eggerthellaceae* genus increased 7-fold which aligns with the known ability of the *Eggerthellaceae* family to metabolize (poly)phenols. The genus has also been positively related to lipid metabolism (85). The functions of *Howardella* and *Faecalibacillus* are poorly known and present new genera to explore in the realm of (poly)phenol metabolism. The only genus to decrease with Aronia was *Bifidobacterium*, with losses approximately 4.7-fold compared to control juice. *Bifidobacterium* species have been previously found to metabolize anthocyanins, which suggests that a HFD may compromise the metabolism of anthocyanins by reducing Bifidobacterium (86).

The influence of HFD was also seen in an analysis of the metabolomic profiles. Profiles from week two and week four show major shifts in metabolic activity regardless of the donor microbiome or juice (Supplementary Figure S4). A comparison of the top discriminating features using hierarchical clustering between weeks two and four showed strong grouping pre- and post-HFD introduction. Significant changes were observed indicating a large-scale shift in metabolic activity due to the HFD and the associated inflammatory stimulus. A closer look at specific discriminating metabolites revealed that sphingomyelin was upregulated after the HFD introduction. Sphingolipids are important components of cellular membranes and are signaling molecules important in the regulation of inflammatory pathways (87, 88). Although definitive clarity on the effects of sphingolipids on inflammation requires additional study, initial results have demonstrated tumor necrosis factor-dependent increases in sphingomyelin (89). Upregulation of sphingomyelin induces production of sphingomyelin-1-phosphate through the action of the kinases Sphk1 and Sphk2 in the sphingolipid-to-glycerolipid pathway (90). Increases in sphingomyelin-1-phosphate have also been associated with immune recruitment, lymphocyte activity, inflammation and progression of inflammatory diseases and cancers (91). Reduced sphingolipids were previously shown to increase intestinal inflammation in irritable bowel disease subjects though interestingly, host-derived sphingolipids were increased systemically (88). Therefore, it is possible that the upregulation of sphingomyelin we detected was an early stress response to increased dietary fat load, mediated by elevated LPS-stimulation of TLR4 and proinflammatory activation.

After the HFD introduction, metabolomic profiling indicated the continued influence of Aronia supplementation. However, the donor impact was stronger with high-fat diet than with normal diet and more donor-specific effects were discovered. Due to multiple strong effectors, examination of the metabolomic profiles show little separation by donor or by juice. Yet, an analysis of metabolomic profiles for each donor indicated different characteristics based on juice treatment. This provides evidence that microbiota from different donors interact uniquely with the polyphenol-rich Aronia fruit juice leading to disparate metabolomic profiles. A focused analysis at 8-weeks was completed and indicated donor and juice specific metabolites upregulated in the mouse models with Aronia supplementation again resulting in an increase in PCs (Figure 8B). To determine observed PC increases were influenced by alterations in microbial makeup or metabolic interactions with the polyphenolic compounds found in Aronia, TMAO was isolated and quantified. TMAO has been shown to be a biomarker for increased risk of cardiovascular disease and other chronic inflammatory diseases and is formed in the liver through the oxidation of TMA by FMO3 (92). TMA can only be generated by a subset of gut microbiota that possess cutC, a glycyl radical enzyme. The cleavage of choline by cutC forms TMA which can then be transported across the lumen. Our data indicates that at week 8, Aronia treated mice had a significantly lower TMAO concentration than control mice (Figure 9). Further analysis of the Aronia treated mice revealed that TMAO concentrations decreased more dramatically in the HI mice than in the LO mice showing a shift in microbial makeup presumably resulting in a decrease in microbes possessing cutC. This analysis indicated that both microbial makeup and phenolic compound availability had an impact on TMAO, and likely PC, concentrations.

While demonstrating several important gut microbiome and metabolic impacts of Aronia juice in humanized mice, we also acknowledge that our study has limitations. Our experiment was set up as a pilot study to demonstrate proof of concept demonstrating gut microbiota dependent impacts linked to inflammation phenotype. As a result, the limited number of mice in our study was dependent on litter sizes. While we demonstrated several key findings, we acknowledge that a greater sample size will be needed to further explore additional impacts. Similarly, both human gut microbiome donors were female, and our study may reflect sex-specific metabolic differences. Sexual dimorphism is present in diet, nutrient metabolism, and gut microbiome composition (93). Thus, our findings need to be confirmed in males and future research incorporating male and female gut microbiota donors is needed to evaluate whether there are differential responses to Aronia fruit juice supplementation.

In this study, we were able to successfully humanize a second-generation of germ-free mice inoculated with stool from human donors with different systemic inflammatory profiles, the results of which were reflected in disparate microbiota and metabolomic profiles. This allowed for a comparison of polyphenol-rich juice treatment, consisting of *Aronia melanocarpa* fruit juice between two distinct mouse populations. Using 16S rRNA sequencing and mass spectrometry analysis, donor specific microbial communities and metabolites demonstrated distinct responses to juice treatment and the subsequent introduction of an inflammatory stimulus through a HFD introduction. Importantly, Aronia juice offered protective effects against HFD that were microbiome dependent. Metabolomic responses were centered around an increase in the phosphatidylcholine with juice supplementation and sphingomyelin with the introduction of HFD. Further research in should consider replication with additional stool donors for better generalizability.

## Data availability statement

The datasets presented in this study can be found in online repositories. The names of the repository/repositories and accession number(s) can be found at: https://www.ncbi.nlm.nih.gov/, PRJNA596000, https://www.ncbi.nlm.nih.gov/, PRJNA906115, https://www.metabolomicsworkbench.org, ST004409.

## Ethics statement

The studies were conducted in accordance with the local legislation and institutional requirements. The studies involving humans were approved by the Montana State University Institutional Review Board. The participants provided their written informed consent to participate in this study. The animal study was approve by the Montana State University IACUC.

## Author contributions

CY, SWa, ZM, BB, and MM: conceptualization. MM, CY, SWa, BB, and JP: methodology. SWi and JP: software, formal analysis, data curation, writing—original draft preparation, and visualization. SWi, JP, and HF: investigation. SWa, ZM, BB, and MM: resources. SWi, JP, CY, SWa, BB, and MM: writing—review and editing. SWi: project administration. SWa and MM: supervision. CY, SWa, ZM, BB, and MM: funding acquisition. All authors contributed to the article and approved the submitted version.

## Funding

This research was supported by Montana State University Research Initiative 51040-MSUR12015-03 and USDA-NIFA 2017-67018-26367. Funding for the Montana State Mass Spectrometry Facility used in this publication was made possible in part by the MJ Murdock Charitable Trust, the National Institute of General Medical Sciences of the National Institutes of Health under Award Numbers P20GM103474 and S10OD28650, and the MSU Office of Research and Economic Development. Funding sources were not involved in the design, data collection and analysis, and writing of the manuscript.

## Conflict of interest

The authors declare that the research was conducted in the absence of any commercial or financial relationships that could be construed as a potential conflict of interest.

## Publisher’s note

All claims expressed in this article are solely those of the authors and do not necessarily represent those of their affiliated organizations, or those of the publisher, the editors and the reviewers. Any product that may be evaluated in this article, or claim that may be made by its manufacturer, is not guaranteed or endorsed by the publisher.
