# Supplementary material for: Metabolic impact of polyphenol-rich aronia fruit juice mediated by inflammation status of gut microbiome donors in humanized mouse model
Source: Front Nutr. 2023 Sep 1;10:1244692. doi: 10.3389/fnut.2023.1244692 (PMC10505616; doi:10.3389/fnut.2023.1244692)
Supplement: Supplementary file 1 [file Data_Sheet_1.docx]

Supplementary Material

Metabolic Impact of Polyphenol-rich Aronia Fruit Juice Mediated by Inflammation Status of Gut Microbiome Donors in Humanized Mouse Model

Stephanie M.G. Wilson^1^†, Jesse T. Peach^2^†, Hunter Fausset^2^, Zachary T. Miller^5^, Seth T. Walk^3^, Carl J. Yeoman^4^, Brian Bothner^2^, Mary P. Miles^1^*

† These authors share first authorship.

*** Correspondence:** Corresponding Author: [mmiles@montana.edu](mailto:mmiles@montana.edu)

# Supplementary Data

No supplementary data is provided.

# Supplementary Figures and Tables

## Supplementary Figures
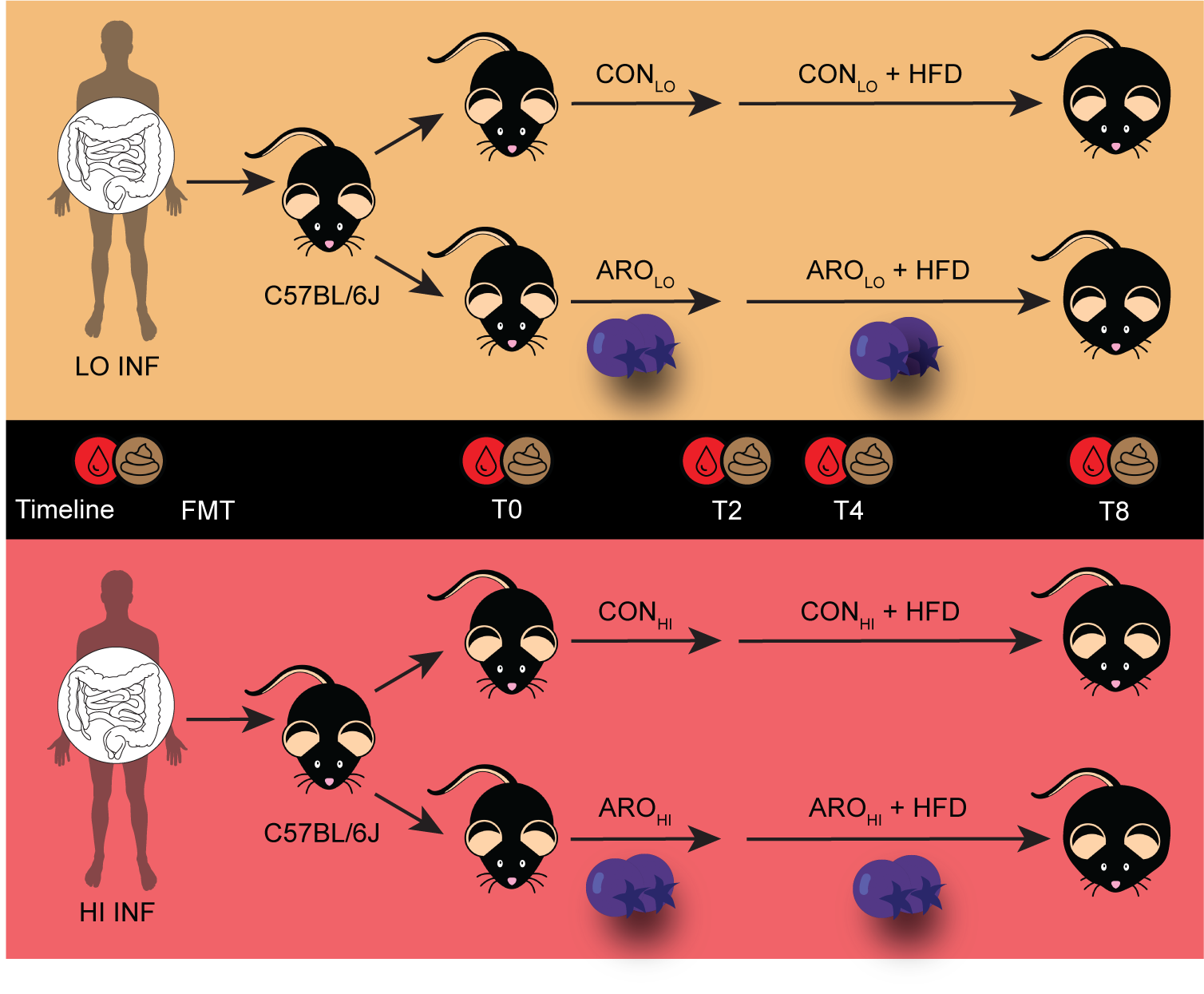
Supplemental Figure 1. Outline of Key Events and Sample Collection. Adults (n=40) with a BMI 27-36 kg/m^2^ self-collected stool samples and underwent testing for anthropometric, metabolic, and proinflammatory measures. Germ-free female C57BL/6J mice were inoculated with stool from 1 of 2 human donors with low or high chronic low-grade inflammation. Second-generation mice received Aronia (ARO) juice (ARO_LO_, = 3, ARO_HI_, n=5), or a sugar-matched control (CON) for 2 weeks (CON_LO_, n=3, CON_HI_, n = 3). After a 2-week juice acclimation period, mice switched from standard diet to a high-fat diet (HFD) for 6 weeks but resumed their original juice treatment. Blood and fecal samples were assessed at baseline, two weeks (T2), four weeks (T4), and eight weeks (T8) for gut microbial composition (by Illumina MiSeq amplicon sequencing) and serum metabolites (by LCMS).


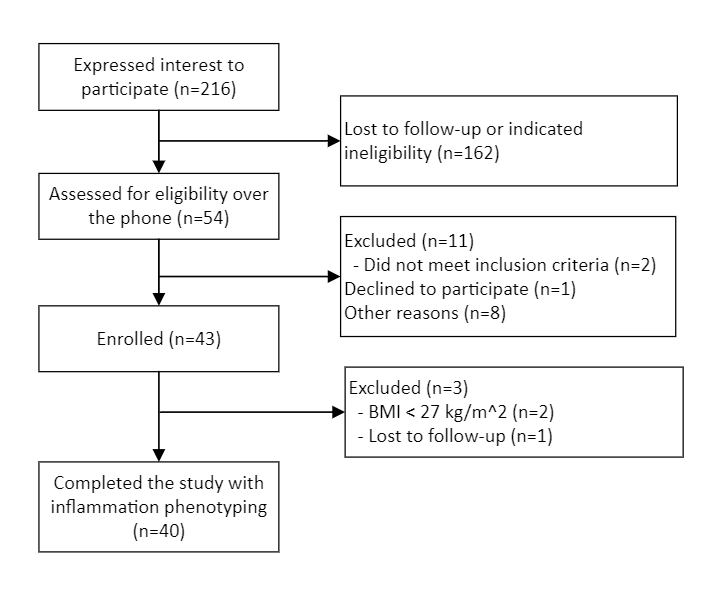


**Supplemental Figure 2**. Human consort diagram. Flyers and e-mails were used to recruit interested individuals. Over two hundred interested individuals contacted the research team, who sent them additional information about the study in return. Fifty-four individuals completed a phone screening with the same researcher. Forty-three individuals met the requirements and were enrolled to participate. Of these, two individuals were found to be ineligible on the first visit and one individual was unable to be contacted. Forty individuals completed the remaining blood work portion of the study and were able to have their serum inflammatory profile phenotyped.


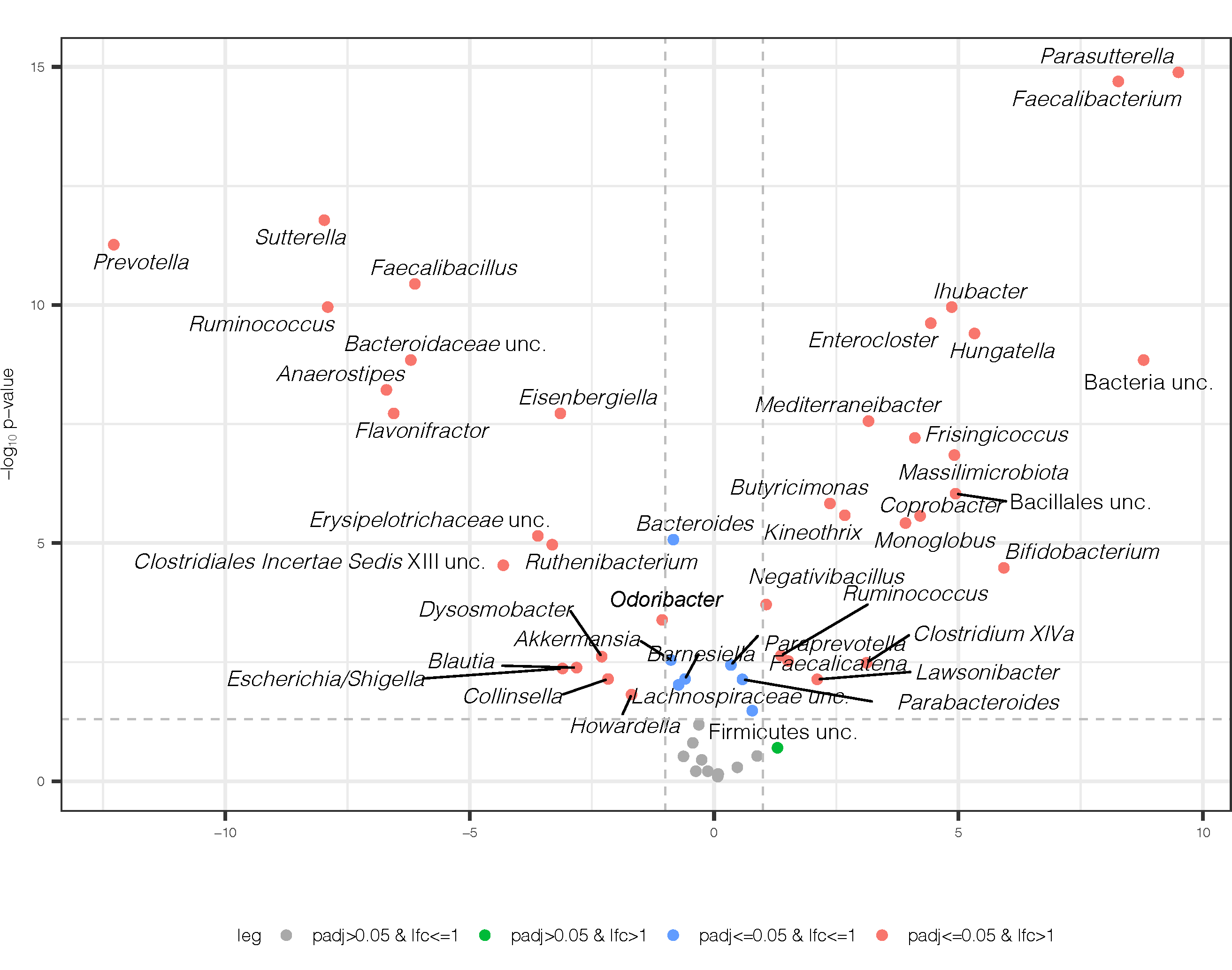


**Supplemental Figure 3**. Differential bacterial genera by donor at baseline as detected by LinDA. Volcano plots show differential genera in LO INF mice compared to HI INF mice as the reference. Abbreviations: padj, Benjamini-Hochberg adjusted p-value; lfc, log fold change.


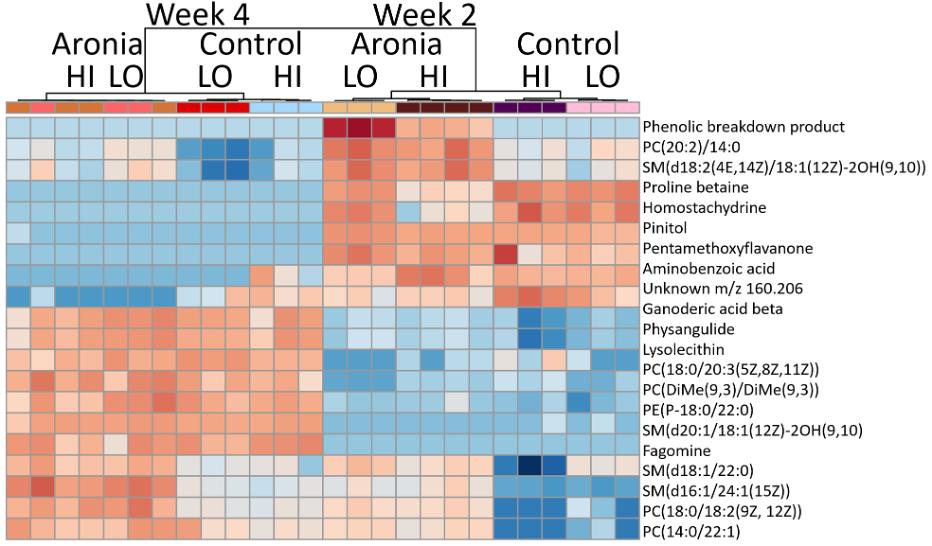


**Supplemental Figure 4**. Introduction of the HFM had a major impact on metabolomic profiles of mice regardless of donor or experimental treatment. Hierarchical clustering indicated the importance of the HFD introduction from week two to week four. Clustering still occurs by both donor and experimental treatment within each time point.

## Supplementary Tables

**Supplementary Table 1**. Key Resources Table.

| **REAGENT or RESOURCE** | **SOURCE** | **IDENTIFIER** |
| --- | --- | --- |
| **Biological Samples** |  |  |
| Human - Blood | Nutrition Research Laboratory - Montana State University | IRB#MM02116-FC |
| Mouse - Blood | Animal Resource Center - Montana State University | IACUC#2017-23 |
| Human - Stool | Nutrition Research Laboratory - Montana State University | IRB#MM02116-FC |
| Mouse - Stool | Animal Resource Center - Montana State University | IACUC#2017-23 |
| **Chemicals** |  |  |
| Acetone | Fisher Scientific | A949 |
| Methanol | Fisher Scientific | A452 |
| Water (UHPLC-MS) | Fisher Scientific | W6 |
| Formic Acid | Fluka | 94318 |
| Acetonitrile | Fisher Scientific | A998 |
| Ammonium Formate | Sigma-Aldrich | 78314 |
| Trimethylamine-oxide | Sigma-Aldrich | 317594 |
| Anthocyanin standards | Sigma-Aldrich | SKU#PHL80577-10MG |
| Sodium trimethylsilylpropanesulfonate | Sigma-Aldrich | **178837** |
| **Deposited Data** |  |  |
| Gut Microbial DNA sequencing - Human | Sequence Read Archive | PRJNA596000 |
| Gut Microbial DNA sequencing - Mice | Sequence Read Archive | PRJNA906115 |
| Serum Metabolomics - Mice and Human | National Metabolomics Data Repository, www.metabolomicsworkbench.org | ST004409 |
| **Dietary Components** |  |  |
| *Aronia melanocarpa* juice | Western Agricultural Research Center, Corvallis, Montana | NA |
| Standard Chow, autoclavable | LabDiet | Cat#50A3 |
| High-Fat Diet, irradiated | Teklad | Cat#TD.96132 |
| **Experimental Organisms** |  |  |
| C57BL/6J mice | The Jackson Laboratory | Cat#000664 |
| **Software and Algorithms** |  |  |
| R Statistical Computing Software | Posit, https://www.posit.co/ | v. 4.2.2 |
| MOTHUR | https://www.mothur.org/ | v.1.44.3 |
| MetaboAnalyst | metaboanalyst version 4.0 | v. 4.0 |
| MZmine | MZmine version 2.53 | v. 2.53 |
| msconvert | ProteoWizard |  |
| SIRIUS | SIRIUS version 4.0.1 | v. 4.0.1 |
| Chenomx | NMR Suite 7.6 |  |

**Supplementary Table 2**. Discriminating metabolites. ID levels are based on the manuscript “Identifying Small Molecules via High Resolution Mass Spectrometry: Communicating Confidence”, by Schymarski et al.

| **Week 0** | | | | | | |
| --- | --- | --- | --- | --- | --- | --- |
| **m/z** | **Compound** | **Adduct** | **RT (mins)** | **Upregulation** | **p-value (t-test)** | **ID Level** |
| 122.025 | Cysteine | M+H^+^ | 4.7 | HI Donor | 1.90E-02 | L1 |
| 132.075 | 3-Hydroxyproline | M+H^+^ | 5.0 | HI Donor | 2.40E-02 | L3 |
| 146.165 | Spermidine | M+H^+^ | 9.1 | HI Donor | 2.60E-02 | L3 |
| 148.038 | 6-Methylaminopurine | M+H^+^ | 4.8 | HI Donor | 7.00E-03 | L2A |
| 180.147 | Dimethylaminoethylbenzeneamine | M+H^+^ | 6.1 | HI Donor | 2.00E-03 | L3 |
| 203.152 | Asymmetric dimethylarginine | M+H^+^ | 3.6 | HI Donor | 3.00E-03 | L2A |
| 231.080 | 1-Methoxyphenanthrene | M+H^+^ | 2.3 | HI Donor | 3.10E-02 | L3 |
| 259.075 | Serylmethionine | M+H^+^ | 3.0 | HI Donor | 7.00E-03 | L3 |
| 259.076 | 4'-Thiothymidine | M+H^+^ | 3.1 | HI Donor | 1.00E-03 | L3 |
| 275.102 | Phenylalanylserine | M+H^+^ | 2.0 | HI Donor | 1.30E-02 | L3 |
| 497.315 | Pregn-5-ene-3,16,20-triol 3-glucoside | M+H^+^ | 1.9 | LO Donor | 2.90E-02 | L3 |
| 498.898 | Indolepyruvate | M+H^+^ | 4.4 | LO Donor | 3.20E-02 | L3 |
| 545.342 | Milbemycin beta 1 | M+H^+^ | 3.9 | LO Donor | 2.70E-02 | L3 |
| 556.852 | Unknown | NA | 4.4 | LO Donor | 0.050 | NA |
| 556.854 | Unknown | NA | 4.2 | LO Donor | 9.00E-03 | NA |
| 627.533 | 7-docosa-dienoyloxyheptadecanate | M+H^+^ | 6.9 | LO Donor | 4.90E-02 | L3 |
| 629.547 | DG(18:1n9/0:0/20:2n6) | M+H^+^ | 4.0 | LO Donor | 1.10E-02 | L3 |
| 685.506 | DG(20:4/0:0/17:0) | M+H^+^ | 5.0 | LO Donor | 3.30E-02 | L3 |
| 1019.694 | TG(22:6/20:5/22:6) | M+H^+^ | 6.5 | HI Donor | 3.10E-02 | L3 |
| 1269.828 | Unknown | NA | 5.7 | LO Donor | 3.70E-02 | NA |
| **Week 2** | | | | | | |
| **m/z** | **Compound** | **Adduct** | **RT (mins)** | **Upregulation** | **p-value (ANOVA)** | **ID Level** |
| 101.057 | Unknown | NA | 4.4 | LO Control | 6.10E-04 | NA |
| 104.165 | Unknown | NA | 4.6 | Aronia | 1.20E-03 | NA |
| 144.100 | Homoleucine | M+H^+^ | 6.3 | Control | 8.50E-06 | L2A |
| 150.997 | 5-Hydroxy-2-furate | M+H^+^ | 2.8 | HI Con/LO Aro | 2.60E-03 | L3 |
| 160.184 | 4,4'-Diaminodibutylamine | M+H^+^ | 4.4 | LO Control |  | L3 |
| 188.074 | Indoleacrylic acid | M+H^+^ | 5.5 | LO Aronia | 1.40E-05 | L3 |
| 197.001 | Phenolic breakdown product | M+H^+^ | 3.3 | Aronia | 7.20E-04 | L4 |
| 217.067 | 1-Hydroxypyrene | M+H^+^ | 3.0 | LO Aronia | 8.00E-04 | L2A |
| 331.020 | Pyrroloquinoline quinone | M+H^+^ | 2.9 | HI Con/LO Aro | 1.80E-04 | L3 |
| 411.142 | Phenolic breakdown product | M+H^+^ | 2.9 | LO Aronia | 8.40E-04 | L4 |
| 511.047 | Unknown | NA | 4.0 | HI Con/LO Aro | 1.20E-06 | NA |
| 520.338 | LysoPC(18:2/0:0) | M+H^+^ | 6.5 | Aronia | 8.70E-04 | L2A |
| 568.337 | LysoPC(20:3/0:0) | M+H^+^ | 6.4 | Aronia | 7.30E-04 | L2A |
| 758.568 | PC(20:2/14:0) | M+H^+^ | 5.8 | Aronia | 2.10E-04 | L2A |
| 759.569 | SM(18:2/18:1) | M+H^+^ | 5.9 | Aronia | 2.90E-04 | L3 |
| 785.650 | SM(16:1/24:1) | M+H^+^ | 6.1 | Aronia | 1.80E-06 | L2A |
| 786.650 | PC(18:1/18:1) | M+H^+^ | 5.7 | Aronia | 7.40E-05 | L2A |
| 787.665 | SM(18:1/22:0) | M+H^+^ | 6.6 | Aronia | 5.80E-07 | L2A |
| 788.661 | PC(18:0/18:1) | M+H^+^ | 5.8 | Aronia | 2.00E-05 | L2A |
| 811.600 | SM(8:0;2O/34:3) | M+H^+^ | 5.7 | LO Control | 4.90E-04 | L2A |
| **Week 8** | | | | | | |
| **m/z** | **Compound** | **Adduct** | **RT (mins)** | **Upregulation** | **p-value (ANOVA)** | **ID Level** |
| 101.057 | Senoic acid | M+H^+^ | 4.4 | Control | 2.20E-06 | L3 |
| 160.080 | 6-Methoxyqquinoline | M+H^+^ | 2.8 | Control | 2.80E-04 | L2A |
| 160.184 | 4,4'-Diaminodibutylamine | M+H^+^ | 4.4 | Control | 2.40E-05 | L3 |
| 160.206 | Unknown | NA | 4.5 | Control | 5.40E-06 | NA |
| 184.056 | Tryptophol | M+H^+^ | 2.3 | HI Aronia | 5.40E-04 | L3 |
| 215.124 | Undecanedoic acid | M+Na^+^ | 2.1 | LO Con/HI Aro | 8.60E-05 | L4 |
| 217.067 | 1-Hydroxypyrene | M+H^+^ | 3.0 | Aronia | 3.90E-04 | L2A |
| 270.139 | Capsiamide | M+H^+^ | 1.9 | LO Con/HI Aro | 5.80E-04 | L3 |
| 283.148 | 4-Acetyl-6-tert-butyl-1,1-dimethyllindane | M+H^+^ | 1.9 | LO Con/HI Aro | 2.90E-04 | L3 |
| 329.005 | 5(3'hydroxyphenyl)-valerate-3'O-sulfate | M+H^+^ | 2.7 | HI Donor | 1.60E-04 | L3 |
| 439.170 | Phenolic breakdown product | M+H^+^ | 1.9 | HI Aronia | 1.20E-06 | L4 |
| 439.170 | Phenolic breakdown product | M+H^+^ | 2.5 | HI Aronia | 1.30E-05 | L4 |
| 457.014 | Shoyuflavone C | M+H^+^ | 1.9 | HI Control | 1.90E-04 | L3 |
| 758.568 | PC(20:2/14:0) | M+H^+^ | 5.8 | LO Aronia | 6.10E-04 | L2A |
| 759.569 | SM(18:2/18:1) | M+H^+^ | 5.9 | LO Aronia | 5.80E-04 | L3 |
| 761.587 | SM(18:1/18:1) | M+H^+^ | 6.0 | LO Aronia | 1.70E-04 | L3 |
| 784.581 | PC(18:1/18:2) | M+H^+^ | 5.8 | LO Aronia | 8.10E-06 | L2A |
| 785.650 | SM(16:1/24:1) | M+H^+^ | 5.8 | Aronia | 1.60E-05 | L2A |
| 786.650 | PC(18:1/18:1) | M+H^+^ | 5.7 | Aronia | 7.50E-05 | L2A |
| 830.564 | PC(PGD1/16:0) | M+H^+^ | 5.6 | HI Aronia | 5.80E-04 | L3 |
